# Integration of phospho-signaling and transcriptomics in single cells reveals distinct Th17 cell fates

**DOI:** 10.1016/j.celrep.2025.116006

**Published:** 2025-07-16

**Authors:** Seth D. Fortmann, Awalpreet S. Chadha, Blake F. Frey, Asif Elahi, Vidya Sagar Hanumanthu, Shanrun Liu, Andrew Goldsborough, P. Brent Ferrell, Maria B. Grant, Casey T. Weaver, Robert S. Welner

**Affiliations:** 1 Medical Scientist Training Program (MSTP), University of Alabama at Birmingham (UAB), Birmingham, AL, USA; 2 Department of Ophthalmology, UAB, Birmingham, AL, USA; 3 Department of Medicine, Division of Hematology/Oncology, O’Neal Comprehensive Cancer Center, UAB, Birmingham, AL, USA; 4 Department of Pathology, UAB, Birmingham, AL, USA; 5 Flow Cytometry and Single Cell Core Facility, Institutional Research Core Program, UAB, Birmingham, AL, USA; 6 RNAssist Limited, Cambridge, UK; 7 Department of Medicine, Division of Hematology/Oncology, Vanderbilt University Medical Center, Nashville, TN, USA; 8 These authors contributed equally; 9 Lead contact

## Abstract

Single-cell RNA sequencing (scRNA-seq) provides the resolution and scale necessary to identify transcriptional programs but fails to capture post-transcriptional information critical to decipher signaling networks and cellular states. We present Vivo-seq, an innovative platform that integrates scRNA-seq and intracellular cellular indexing of transcriptomes and epitopes by sequencing following cellular fixation with a deep eutectic solvent, which preserves multiple domains of biological information beyond RNA transcripts alone. Vivo-seq enables simultaneous capture of both transcriptional and phospho-signaling states in single cells. Applying this platform to developing T helper 17 (Th17) cells, we find that simultaneous phosphorylation of ERK1/2 and c-FOS leads to maximal interleukin-2 (IL-2) and IL-17 production. Furthermore, we show that early IL-2 production imprints developing Th17 cells for enhanced maintenance or cytokine-dependent transdifferentiation during subsequent antigenic stimulation. By integrating transcriptional and phospho-signaling information at single-cell resolution, we identify a hyperactivated Th17 cellular state associated with early IL-2 production that has downstream consequences on functional plasticity.

## In brief

Vivo-seq allows the simultaneous capture of transcription and intracellular phosphorylation in droplet-based scRNA-seq. Using this approach in Th17 differentiation, we identify dual phosphorylation of ERK1/2 and c-FOS as required for maximum IL-2 production and associated with enhanced Th17 maintenance or transdifferentiation depending on subsequent cytokine signals received during antigenic restimulation.

## Graphical Abstract

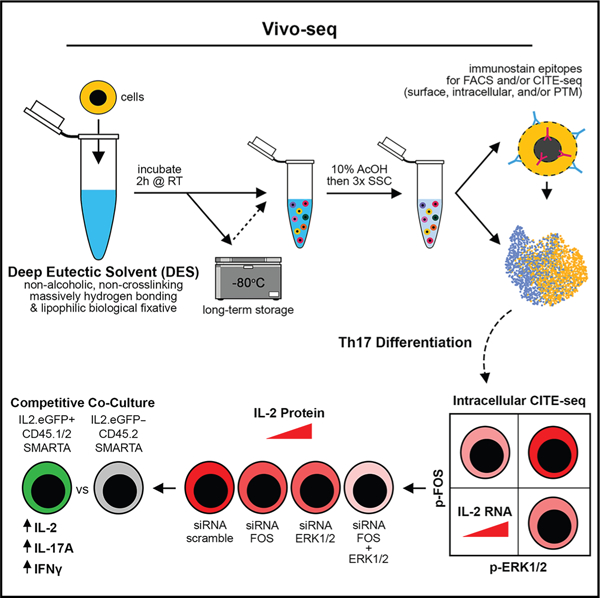

## INTRODUCTION

Intracellular signaling networks mediate the continuous exchange of information to maintain homeostasis and facilitate cellular adaptation to changing environments. Accordingly, the reconstruction of these networks necessitates the concurrent collection of multiple domains of biological data with high resolution and in a massively parallel fashion. To this end, single-cell RNA sequencing (scRNA-seq) provides the required resolution and scale, but RNA alone only partially captures cellular states, and the loss of labile biological information, such as intracellular phosphorylation, remains a core challenge.

Chemical fixation is designed to preserve cellular states and prevent the loss of biological data. The most widely used fixatives are formaldehyde and alcohols, which act through protein cross-linking and dehydration, respectively. However, both of these chemical classes impose undesirable constraints, including the degradation of RNA and/or the skewing of its recovery post-fixation.^1–3^ Deep eutectic solvents (DESs) are a novel class of chemicals with unique properties favorable for next-generation biological fixatives. DESs are composed of a hydrogen bond donor and acceptor, which, when mixed in particular stoichiometric ratios, induces a characteristically large melting point depression and the subsequent formation of a viscous room temperature liquid.^4,5^ A subset of DES’s, including the commercially available product vivoPHIX (RNAssist, Cambridge, UK), rapidly penetrate and fix bacterial, plant, and animal cells, likely through prolific intermolecular hydrogen bonding that displaces water in the hydration shells of biomolecules.^6^ Importantly, DES fixation reportedly allows the long-term stabilization of biomolecules, while weakening cell-to-cell interactions facilitating dissociation post-fixation and long-term storage.^6,7^ Thus, DES-based compounds are an attractive alternative to traditional biological fixatives and can preserve multiple domains of biological data.

Here, we demonstrate that DES fixation allows the simultaneous integration of phospho-signaling and transcriptional information in single cells during T helper 17 (Th17) cell differentiation. We show that DES retains complex cellular morphologies, such as colonic enterocytes, during ultrasonic dissociation, allows long-term stabilization of single-cell information, preserves post-translational modifications (PTMs), and provides sufficient membrane permeabilization for probing intracellular epitopes. With DES fixation, we establish a validated and flexible approach to intracellular CITE-seq (cellular indexing of transcriptomes and epitopes by sequencing), called Vivo-seq, and apply it to study four phosphorylated intracellular proteins during Th17 cell differentiation. We reveal that the concurrent phosphorylation of extracellular signal-regulated kinase 1/2 (ERK1/2) and c-FOS during early Th17 cell differentiation results in a hyperactivated state associated with increased interleukin-2 (IL-2) production and link this with IL-17 output and downstream Th17 cell maintenance and transdifferentiation. These results reveal an unknown association between ERK1/2 and FOS activation, IL-2 production, and subsequent cellular fates in Th17 cells. We anticipate that using Vivo-seq in single-cell technologies will improve the reconstruction of signaling networks and help illuminate how intracellular signaling shapes gene expression and cellular function in health and disease.

## RESULTS

### DES fixation preserves RNA quality and quantity and allows intracellular immunostaining

**Figure 1.**
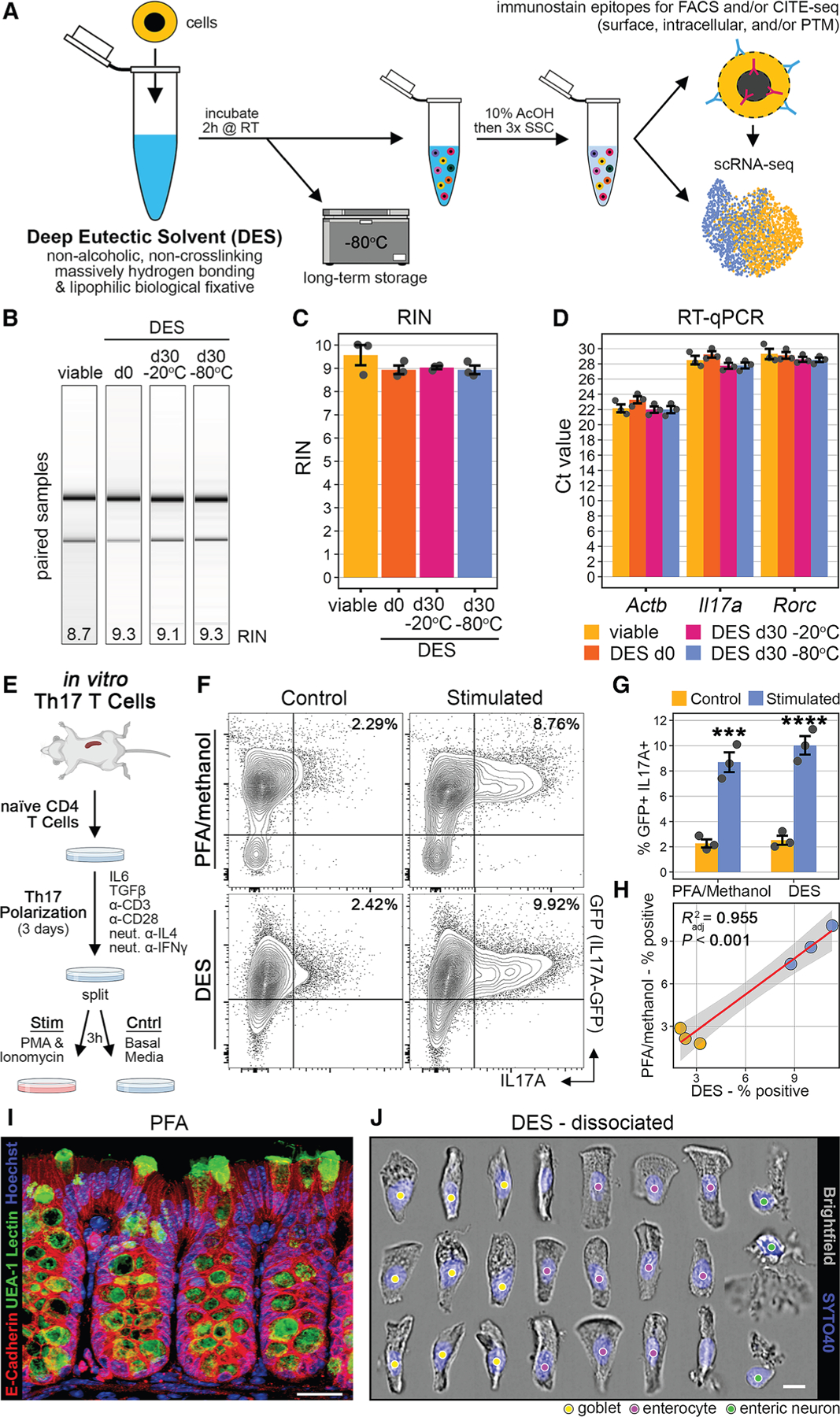
DES fixation allows RNA preservation and intracellular immunostaining (A) Schematic overview of DES fixation and intracellular CITE-seq. AcOH, acetic acid; FACS, fluorescence-activated cell sorting; PTM, post-translational modification; RT, room temperature; SSC, saline sodium citrate. (B) Automated gel electrophoresis for RNA integrity from primary mouse Th17 T cells. The same culture is shown across each time point/condition. (C) RNA integrity number (RIN) from primary mouse Th17 T cells. (D) RT-qPCR from 100,000 FACS-sorted primary mouse Th17 T cells. (E) Schematic overview of *in vitro* conditions for Th17 polarization of mouse naive CD4 T cells. α, anti; Cntrl, control (basal conditions); Neut., neutralizing; PMA, phorbol myristate acetate; Stim, stimulated. (F) Representative flow cytometry plots of stimulated and basal (control) primary Th17 T cells from Il17a-GFP transgenic mice. Top row is standard PFA/methanol, bottom row is DES. (G) Quantification of GFP^+^/IL17a-protein^+^ cells with PFA/methanol versus DES (*n* = 3). (H) Linear regression comparing GFP^+^/IL17a-protein^+^ cells with PFA/methanol versus DES. (I) Confocal microscopy of PFA-fixed mouse colon. Scale bar, 25 μm. (J) Collage of imaging flow cytometry photos from DES fixed and ultrasonically dissociated mouse colon. Scale bar, 7 μm. Yellow circles, goblet cells; magenta circles, enterocytes; green circles, enteric neurons. One-way ANOVA with Dunn’s post hoc test. ***p ≤ 0.001; ****p ≤ 0.0001. Data are represented as mean ± SEM.

We developed an innovative approach using a commercially available DES-based fixative (vivoPHIX) that preserves cellular states with minimum loss of biological information in droplet-based scRNA-seq (Figure 1A). As an overview, cells are immersion fixed in DES for ≥2 h at room temperature, followed either by immediate downstream processing or long-term storage at −80°C. The cells are then briefly treated with 10% glacial acetic acid (AcOH; diluted in DES), which inactivates cellular RNAses for resuspension in aqueous buffer.^8^ The 10% AcOH/DES cell slurry is then transferred to an aqueous solution via an intermediate wash step with excess 3× saline sodium citrate (SSC), and resuspension in 1× SSC for all downstream processing. The cell suspension can then be used for a wide variety of standard analytical techniques.

First, we optimized the transition from DES to aqueous solution in fixed, dissociated bone marrow, which is required for compatibility with standard downstream analytical techniques. Similar to other fixatives, like methanol,^3^ the transition from DES to aqueous buffer is a critical and highly sensitive step where RNAses can become reactivated, leading to acute degradation. We tested three aqueous transition strategies in paired bone marrow samples: (1) treatment with 10% AcOH followed by excess 3× SSC, (2) excess 3× SSC alone, as previously reported for methanol-fixed cells,^3^ and (3) methanol treatment followed by resuspension in a high-salt buffer akin to diluted RNAlater, as recently reported for methanol-fixed cells.^9,10^ With the 10% AcOH to 3× SSC approach, gel electrophoresis revealed intact and undegraded RNA, whereas 3× SSC alone resulted in moderate RNA degradation, and methanol to high salt resulted in severe RNA degradation (Figure S1).

Second, using the AcOH transition strategy, we quantitatively assessed the ability of DES to preserve RNA quality in acutely fixed and long-term stored samples. To this end, we utilized a murine primary culture approach of Th17 T cells,^11^ which allowed for a homogeneous and tightly controlled primary cell population. Briefly, murine naive T cells isolated from the spleen were polarized under Th17 conditions for 3 days. Following this, the cultures were split into viable and DES-fixed groups, and aliquots of the DES-fixed cells were stored at −20°C or −80°C for 1 month to determine the effects of long-term storage. It should be noted that even at −80°C, DES does not freeze. Gel electrophoresis for the visualization of RNA degradation (Figure 1B) and RNA integrity number analysis (Figure 1C) confirmed the ability of DES to preserve RNA quality. We performed RT-qPCR using 100,000 fluorescence-activated cell sorting (FACS)-sorted cells per sample and found no difference in Ct values between groups (Figure 1D), consistent with preserved RNA quantity.

An empirical observation of DES fixation is that it provides sufficient membrane permeabilization for intracellular staining. Therefore, we next assessed the reliability of intracellular staining after DES fixation by comparing it to that of a commercial mixture of paraformaldehyde (PFA)/methanol, the gold standard. Naive T cells isolated from IL17a-GFP transgenic mice (which report GFP as a marker of intracellular IL-17A activity) were cultured under Th17 polarizing conditions followed by a secondary stimulation with phorbol myristate acetate (PMA)/ionomycin in half the cells (Figure 1E). Using Il17a-GFP transgenic mice, we compared flow cytometry quantifications of double-positive GFP^+^/anti-IL17A^+^ cells in identical cultures under stimulated versus control conditions using either DES or standard PFA/methanol. Both methods resulted in a similar recovery of double-positive cells (Figure 1F), which were highly significant compared to paired control cultures (Figure 1G). Importantly, linear regression comparing the double-positive percentage of each culture for DES versus PFA/methanol revealed a robust and highly significant correlation (*R*^*2*^ = 0.955; Figures 1H andS2), demonstrating that intracellular staining with DES is reproducible and reliable compared to standard methods.

Another unique feature of DES fixation is that it preserves cellular morphology and is amendable to mechanical dissociation post-fixation. To demonstrate this, we used mouse colonic epithelium and compared cell morphology from confocal microscopy of PFA-fixed mouse colon to imaging flow cytometry of the DES-dissociated equivalent. In PFA-fixed mouse colon, E-cadherin staining distinguished the colonic epithelium that lined crypts and the interface of the intestinal lumen, while UEA-1 lectin marked mucin-producing goblet cells, with characteristic narrow bases and cup-shaped apical bodies (Figure 1I). Imaging flow cytometry of DES-dissociated colon revealed near-identical morphologies, and subtypes of colonic epithelium were readily identifiable (Figure 1J). Thus, we used a challenging tissue to demonstrate that DES preserves *in vivo* morphology.

### DES preserves phosphorylation and active intracellular signaling

**Figure 2.**
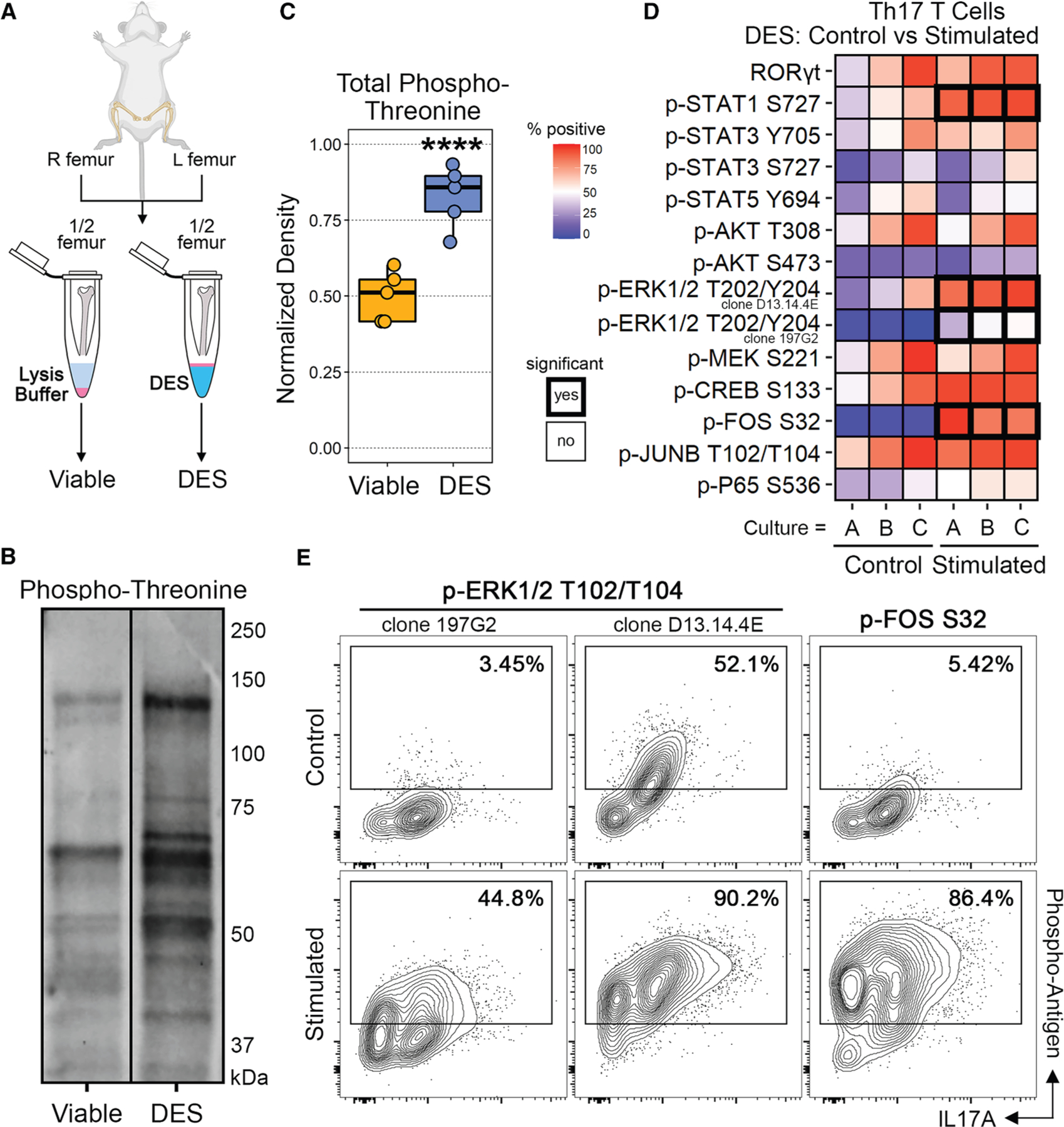
DES preserves phosphorylated epitopes (A) Schematic of experimental design for (B) and (C). Bone marrow from paired femurs was spun out of the bone shafts into either lysis buffer (Viable) or DES. (B) Immunoblot of bone marrow from paired mice for phospho-threonine. Samples were either immediately lysed or fixed with DES prior to lysis. (C) Immunoblot quantifications of phospho-threonine (*n* = 5) from (B). Student’s t test. ****p ≤ 0.0001. (D) Heatmap representation of flow cytometry data using stimulated versus control Th17 T cells showing percent positivity for 12 different phospho-targets. Isotype controls were used for gating, and all cells were fixed with DES. Two-way ANOVA with Holm-Sidak post hoc test. (E) Representative flow cytometry plots from (D) showing two different antibody clones to p-ERK1/2 T204/Y204 (left and center) and p-FOS S32 (right).

We next explored whether DES can preserve *in vivo* phosphorylation, a dynamic process that regulates active intracellular signaling but is generally labile and sensitive to decay.^12^ To this end, we first performed total protein immunoblotting for phospho-threonine from paired murine whole bone marrow samples. Samples were either lysed immediately in the presence of phosphatase inhibitors or fixed in DES prior to lysis (Figure 2A). DES provided robust preservation of phosphorylation across the proteome (Figure 2B), resulting in a highly significant increase in total recovered phosphorylation compared to direct lysis samples (Figure 2C). These data indicate that DES can preserve active intracellular signaling.

Through intracellular CITE-seq (inCITE-seq), DES presents an opportunity to quantify intracellular signaling in scRNA-seq, thereby allowing its parallel integration with transcription. To this end, we assessed the ability of DES to preserve the phosphorylation of individual protein species in single cells using flow cytometry. Utilizing *in vitro* differentiated Th17 cells as described before, we compared the levels of 14 different intracellular targets, including 13 distinct phospho-species, between stimulated and unstimulated cells (Figure 2D). Results between paired cultures and replicates were highly consistent and revealed increased phosphorylation in several species, including significant increases in both antibody clones to p-ERK1/2 (T202/Y204), p-FOS (S32), and non-canonical p-STAT1 (S727), but not canonical p-STAT1 (Y701), which was inhibited by neutralizing interferon-γ (IFN-γ) within the cultures (Figure 2D).^11^ Other phospho-species that showed consistent increasing trends with stimulation but were not significant after correction for multiple comparisons included p-p65 (S536), p-CREB (S133), and non-canonical p-STAT3 (S727) (Figure 2D). Given that PMA, a component of our secondary stimulation (PMA/ionomycin), drives phosphorylation of ERK1/2 via protein kinase C,^13^ we included two different clones to p-ERK1/2 as positive controls, both of which showed significant increases (Figure 2E). Surprisingly, the single phospho-species with the largest increase between stimulated and control cultures was p-FOS (S32), which in total increased from 5.42% to 86.4% (Figure 2E). These data demonstrate that DES preserves phosphorylation and allows for the quantification of individual phosphorylated proteins in single cells using immunostaining.

### Quantification of intracellular phosphorylation with inCITE-seq in DES-fixed cells

**Figure 3.**
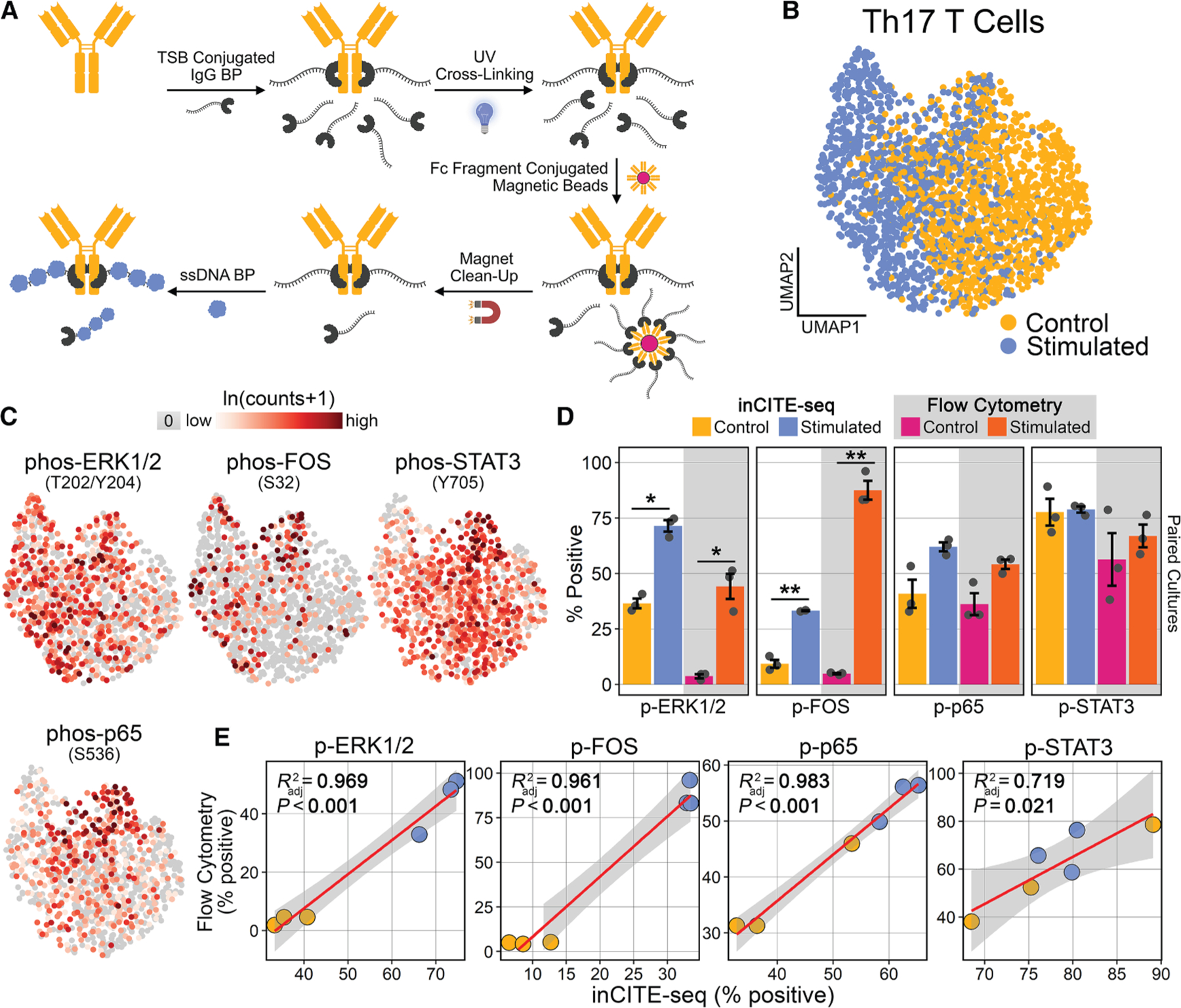
Validation of intracellular CITE-seq for four phosphorylated targets during Th17 stimulation (A) Schematic overview of antibody-oligonucleotide conjugation and preprocessing for inCITE-seq. (B) Uniform manifold approximation and projection (UMAP) of scRNA-seq data from 2,230 cells annotated according to stimulation conditions. (C) UMAP plots of log normalized counts from inCITE-seq for p-ERK1/2 (T204/Y204), p-FOS (S32), p-STAT3 (Y705), and p-p65 (S536). (D) Comparisons of percent positivity for the four phospho-targets using inCITE-seq versus flow cytometry from the same cultures. Positive gating for inCITE-seq was determined as any cell with >0 phospho-target counts after isotype subtraction and natural log (ln) +1 scaling. (E) Linear regressions for the four phospho-targets comparing percent positivity from inCITE-seq versus flow cytometry. One-way ANOVA with Sidak’s multiple comparisons test. *p < 0.05; **p ≤ 0.01. Data are represented as mean ± SEM.

Given that DES preserves PTMs and allows immunostaining of intracellular epitopes, we next sought to develop an optimized, scalable, and modular approach to inCITE-seq in DES-fixed samples (Figure 3A). To this end, we used a photo-crosslinking small peptide (oligo-immunoglobulin G binding protein, oligo-IgG BP) that binds with high affinity and specificity to the Fc region of Igs and conjugated it with a modified TotalSeq-B oligonucleotide (oYo-link). Following incubation in the presence of UV light, single micrograms of antibody can be covalently conjugated with site specificity and high efficiency (Figure 3A). Optimization studies suggested that high levels of background staining resulted from unbound residual IgG BP as well as off-target interactions between the single-stranded DNA (ssDNA) oligonucleotide and endogenous intracellular molecules. To circumvent these issues, we used magnetic beads containing human IgG Fc fragments to remove unbound oligo-IgG BP (Figure S3) and *Escherichia coli* ssDNA BP to block oligonucleotide interactions (Figure S3), as was recently demonstrated.^14^

To evaluate inCITE-seq in DES-fixed cells, we interrogated Th17 cells under stimulated and control conditions and stained four phospho-targets, p-ERK1/2 (T202/Y204), p-FOS (S32), p-STAT3 (Y704), and p-p65 (S536), as well as an isotype control antibody. To minimize technical variation, we controlled for the number of cells during immunostaining, used the same antibody cocktails, and hashtagged the culture replicates into one sample for scRNA-seq. After quality control filtering and dimensionality reduction, 2,230 total cells were recovered, with a clear separation between control and stimulated cells (Figure 3B). RNA recovery was robust and consistent between stimulated and control cells; genes per cell in stimulated versus control (mean ± SD) were 3,026 ± 1,146 versus 2,946 ± 1,251 respectively, and transcripts per cell were 6,644 ± 3,998 versus 6,103 ± 3,837 respectively (Figure S4). For processing of inCITE-seq, isotype control counts were subtracted from each target followed by natural log+1 transformation. Staining distributions for each of the four phospho-species, p-ERK1/2, p-FOS, p-STAT3, and p-p65 (Figure 3C), revealed distinct patterns, some of which had overlapping features with other phospho-targets.

Comparing stimulated and control conditions in each of the three paired cultures revealed a consistent increase in phospho levels for p-ERK1/2, p-FOS, and p-p65, but not p-STAT3 (Figure S5), mirroring our previous findings for the same targets in the same cultures using flow cytometry (Figure 2D). Of note, p-STAT3 was maintained at a high level independent of PMA/ionomycin stimulation, consistent with IL-6-pSTAT3 signaling from the culture media, an essential component of Th17 cell maintenance.^15^ We next determined the percentage of positive staining for inCITE-seq in each of the cultures/conditions for the four phospho-targets (defined as any cell with >0 counts for a target after isotype correction) and compared this to the percentage positive as determined by flow cytometry for the same targets in the same cultures. In agreement, stimulated cultures showed significant increases for p-ERK1/2 and p-FOS with both flow cytometry and inCITE-seq, while neither p-STAT3 nor p-p65 showed significant shifts with either method (Figure 3D). Finally, using linear regressions to compare percent positivity for paired culture/conditions between inCITE-seq and flow cytometry revealed robust and significant positive correlations for all four targets (p-ERK1/2 *R*^*2*^ = 0.969; p-FOS *R*^*2*^ = 0.961; p-p65 *R*^*2*^ = 0.983; p-STAT3 *R*^*2*^ = 0.719; Figure 3E). Similarly, linear regressions for signal magnitude between inCITE-seq and flow cytometry, using log counts and mean fluorescent intensity (MFI), respectively, showed strong positive correlations, except for p-STAT3, which was not significant (p-ERK1/2 *R*^*2*^ = 0.989; p-FOS *R*^*2*^ = 0.914; p-p65 *R*^*2*^ = 0.826; p-STAT3 *R*^*2*^ = 0.379; Figure S5). These data provide definitive validation that phosphorylated proteins can be reliably quantified with inCITE-seq following DES fixation.

Of note, our group is not the first to perform intracellular CITE-seq.^14,16–20^ Of these, most published studies have relied on non-phosphorylated intracellular targets,^14,18,19^ and all utilize a cross-linking chemical fixative. Recently, the Satija laboratory has introduced a high-throughput method for inCITE-seq of phosphorylated epitopes, called Phospho-seq, but this relies on ‘‘bridge integration’’ for inferring RNA levels and does not directly measure RNA and intracellular phosphorylation from the same cell.^16^ The Tape laboratory has also introduced a recent method, called SIGNAL-seq, which generates high-quality, multimodal RNA and intracellular phosphorylation measurements from individual cells but relies on a plate-based approach.^20^ To our knowledge, only one other study has reported a droplet-based single-cell method for multimodal RNA and intracellular phosphorylation measurements.^17^ However, this method lacks internal validation against gold standard methods and requires harsh fixation, resulting in poor RNA recovery in cell lines (<1,000 median genes per cell) and unusable RNA in primary cells (<100 median genes per cell). Our approach preserves RNA integrity, retains phosphorylation, and is directly compatible with commercially available droplet-based single-cell instruments.

### Vivo-seq: Parallel integration of transcription and phosphorylation during Th17 cell development

**Figure 4.**
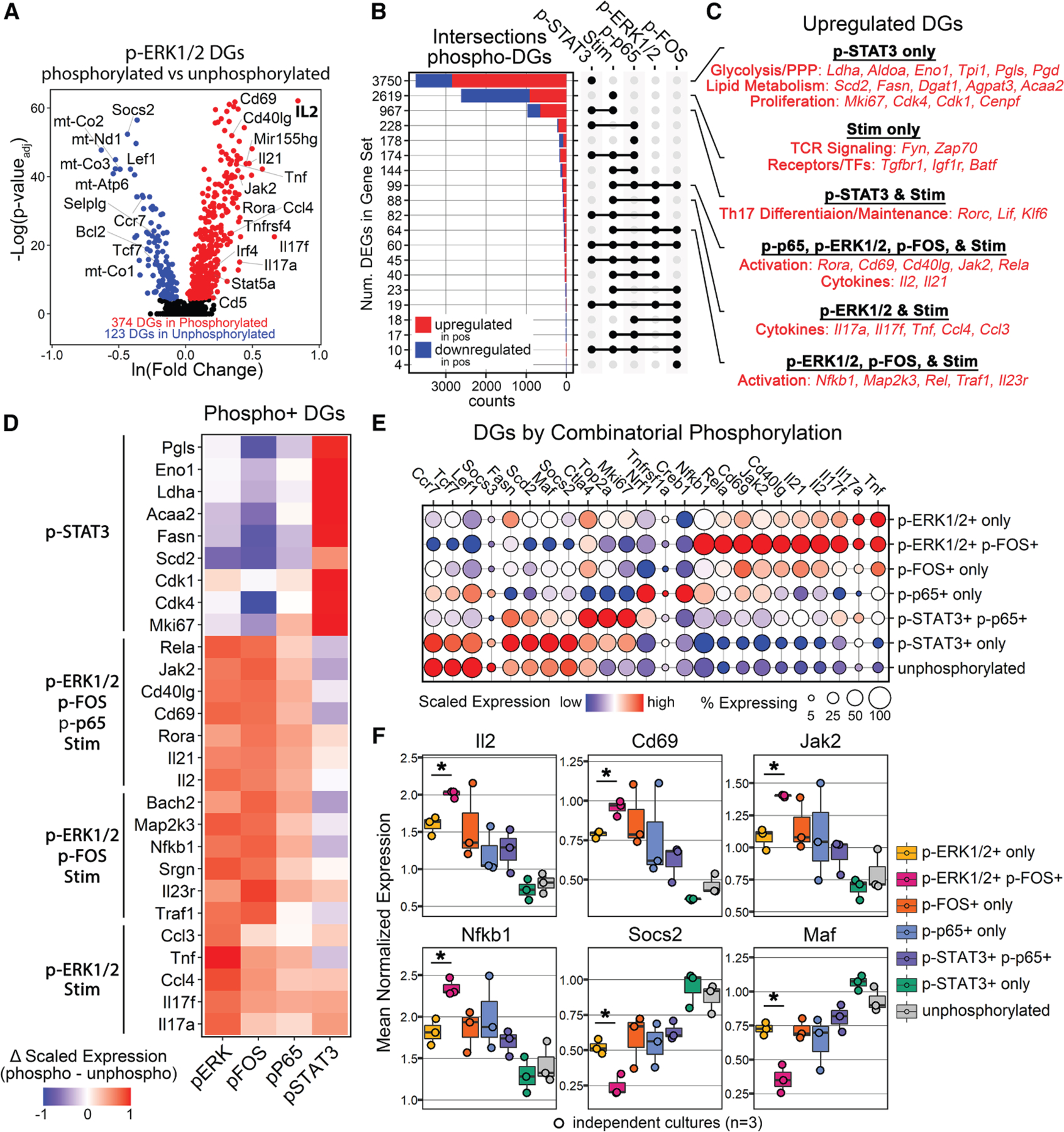
Parallel integration of transcriptomes and phospho-signaling in Th17 stimulation identifies hyperactivated p-FOS^+^/p-ERK^+^ double-positive cells (A) Volcano plot for p-ERK1/2 differentially expressed genes (DGs, phosphorylated p-ERK1/2 versus unphosphorylated p-ERK1/2). (B) Upset plot showing intersections of DG gene sets from each of the four phospho-targets and stimulation. Stimulation refers to stimulated versus unstimulated cells. p-ERK1/2, 497 DGs; p-FOS, 318 DGs; p-p65, 1,013 DGs; p-STAT3, 5,339 DGs; Stimulation (Stim), 4,454 DGs. (C) Selected upregulated DGs from six different gene sets. (D) Heatmap of selected DGs from (C) showing the change in scaled expression between phosphorylated and unphosphorylated cells with respect to each of the four phospho-targets. (E) Combinatorial phosphorylation analysis showing selected top DGs from each combination of phospho-targets. inCITE-seq was performed with two panels of antibodies, and combinations are restricted to the two panels. Panel 1 , p-STAT3 and p-p65; panel 2, p-ERK1/2 and p-FOS. (F) Selected combinatorial phosphorylation DGs from (E) showing culture-wise mean expression. One-way ANOVA with false discovery rate correction. *p < 0.05. Data are represented as mean ± SEM.

Secondary T cell stumulation using PMA/Inomycin functions through phopshorylation of ERK1/2 and calcium influx, respectively.^13,21^ Therefore, as a positive control, we expected that differentially expressed genes (DEGs) from phosphorylated versus unphosphorylated ERK1/2 would be contained within the DEG gene set from stimulated versus unstimulated cells. In agreement, all 497 DEGs identified in p-ERK1/2^+^ cells overlapped with DEGs from the stimulated gene set (Figure S6A). Among the p-ERK1/2^+^ cells, the top upregulated DEGs included several critical genes involved in Th17 cell activation, including *Il17a*, *Il17f*, *Tnf*, *Il2*, *Cd40lg*, *Tnfrsf4*, and *Cd69*, among others (Figures 4A and S6A).^22^ Downregulated genes were enriched for mitochondrial transcripts as well as markers of resting T cells, including *Tcf7*, *Ccr7*, and *Sell* (Figure 4A).^23^ *Il2* was the most highly upregulated gene in p-ERK1/2^+^ cells (Figure 4A), consistent with its central role in early T cell activation,^24^ and Pearson correlation analysis demonstrated a positive relationship between p-ERK1/2 levels and *Il2* gene expression (Figure S6B).

Next, to determine whether specific phosphorylation events were associated with discrete gene sets during Th17 cell activation, such as cytokine production, we computed DEGs for each of the four phospho-targets (Figures S6C–S6F) and compared the intersections of these DEGs with those from stimulated versus unstimulated cells. In total, 22 unique gene sets were identified. The largest gene set was specific to p-STAT3 (Figure 4B), with the most upregulated DEG being *Lgals1*, a recently described direct target of STAT3.^25^ Consistent with prior reports,^26,27^ additional upregulated DEGs in the p-STAT3 gene set included proliferation markers (*Mki67*, *Cdk4*, *Cdk1*, *Cenpf*), lipid metabolism mediators (*Scd2*, *Fasn*, *Dgat1*, *Agpat3*, *Gpat4*, *Acaa2*, *Acot7*, *Fabp5*), and glycolysis and pentose phosphate pathway genes (*Ldha*, *Aldoa*, *Eno1*, *Tpi1*, *Pgls*, *Pgd*) (Figures 4C and 4D). Interestingly, the pSTAT3 gene set did not include markers of T cell activation or cytokine production. The second largest gene set was specific to stimulation only (Figure 4B) and included several ribosomal genes as well as T cell receptor (TCR) signaling proteins (*Fyn*, *Zap70*), cytokine/growth factor receptors (*Tgfbr1*, *Igf1r*), and the transcription factor *Batf* (Figures 4C and 4D). The largest shared gene set was between p-STAT3 and stimulation (Figure 4B) and included key genes involved in Th17 cell differentiation and maintenance such as *Rorc*, *Lif*, and *Klf6*^28,29^ (Figures 4C and 4D). Finally, the gene set shared between p-ERK1/2 and stimulation contained several prominent cytokines and chemokines like *Il17a*, *Il17f*, *Tnf*, *Ccl3*, and *Ccl4* (Figures 4C and 4D), whereas the gene set shared between p-p65, p-ERK1/2, p-FOS, and stimulation contained mostly markers of T cell activation such as *Il2*, *Cd69*, *Cd40lg*, *Jak2*, and *Rela* (Figures 4C and 4D).^22^

We next examined whether specific combinations of phosphorylation events were associated with unique transcriptional states during Th17 cell activation. To this end, we annotated cells as either unphosphorylated, singly phosphorylated, or doubly phosphorylated for each of the two panels of antibodies used in our inCITE-seq experiment (panel 1: p-STAT3 and p-p65; panel 2: p-ERK1/2 and p-FOS). We computed DEGs for each phospho combination and compared their gene expression across combinatorial groups, revealing distinct patterns in transcription (Figure 4E). Compared to p-ERK1/2 only or p-FOS only, p-ERK/p-FOS double-positive cells showed the highest expression of Th17 activation genes, including *Il17f*, *Il2*, *Il21*, *Cd40lg*, *Cd69*, *Mir155hg*, *Jak2*, *Rela*, and *Nfkb1* (Figure 4E).^22^ Similarly, the highest expression of proliferation genes, like *Mki67* and *Top2a*, were seen in p-STAT3/p65 double-positive cells (Figure 4E). p-STAT3-only cells were uniquely enriched in lipid metabolism genes (Figure 4E). Unphosphorylated cells expressed the highest levels of resting markers, like *Ccr7*, *Tcf7*, and *Lef1* (Figure 4E).^23^ Comparing mean expression for each phospho-combination across the three culture replicates revealed a significant increase in several of the aforementioned activation markers in p-ERK/p-FOS double-positive cells compared to p-ERK1/2 alone, including *Il2*, *Cd69*, *Jak2*, and *Nfkb1* (Figure 4F). Furthermore, p-ERK/p-FOS double-positive cells showed significant decreases in levels of suppressive mediators, like Maf and Socs2, compared to p-ERK1/2 alone (Figure 4F).^30,31^ These data suggest that p-ERK/p-FOS double-positive cells possess a unique gene expression program associated with hyperactivation in Th17 cells.

### Combined phosphorylation of ERK1/2 and c-FOS is required for maximum IL-2 expression in Th17 cells

**Figure 5.**
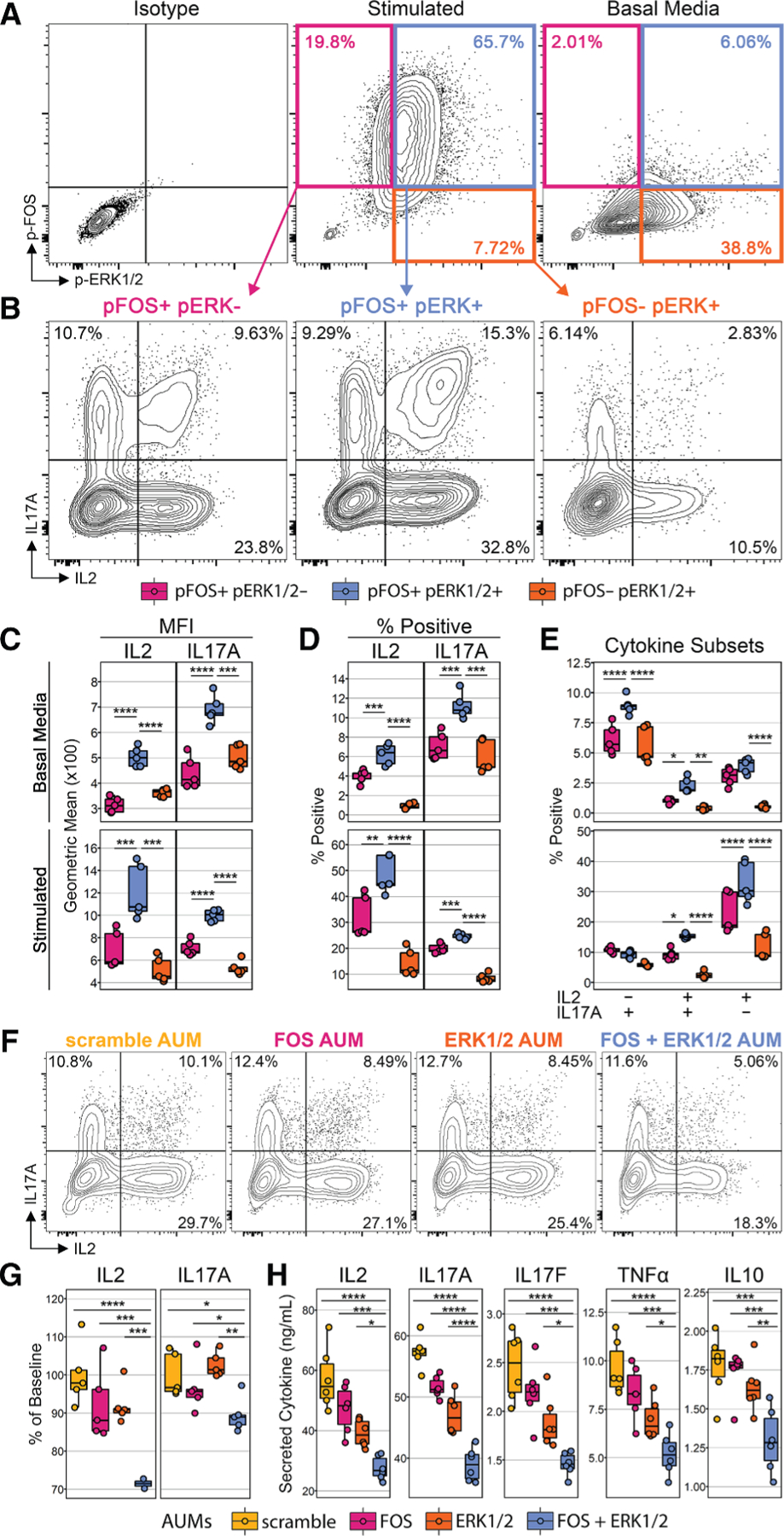
Combined phosphorylation of p-FOS and p-ERK1/2 is required for maximum IL-2 expression in Th17 cells (A–E) Flow cytometry from Th17 T cells (*n* = 5 independent cultures). (A) Representative p-FOS and p-ERK1/2 staining in PFA/methanol-fixed cells showing gating of three populations: p-FOS^+^ p-ERK^−^ (magenta), p-FOS^+^ p-ERK^+^ (blue), and p-FOS^−^ p-ERK^+^ (orange). Isotype control, basal Th17 media, and PMA/ionomycin-stimulated cultures are shown. (B) Representative IL-2 and IL-17A staining of p-FOS^+^ p-ERK^−^, p-FOS^+^ p-ERK^+^, and p-FOS^−^ p-ERK^+^ populations from PMA/ionomycin-stimulated cultures are shown. (C and D) Total IL-2 and IL-17A (C) mean fluorescent intensity (MFI) and (D) percent positivity in p-FOS/p-ERK subsets from basal media and stimulated cultures. One-way ANOVA with Holm-Sidak post hoc test. (E) Percentage of IL-2/IL-17A co-expressing cells in p-FOS/p-ERK subsets from basal media and stimulated cultures. One-way ANOVA with Holm-Sidak post hoc test. (F and G) Flow cytometry from Th17 T cells (*n* = 5 independent cultures) treated with either scramble AUM (yellow); Fos AUM (magenta); Mapk1 and Mapk3 AUMs (orange); or Fos, Mapk1, and Mapk3 AUMs (blue). (F) Representative IL-2 and IL-17A staining from the four AUM treatment groups. (G) IL-2 and IL-17A percent positivity from the four AUM treatment groups, shown as percentage of scramble (baseline). One-way ANOVA with Holm-Sidak post hoc test. (H) Cytokine secretion from Th17 T cells measured in culture supernatant using Legendplex bead array (*n* = 5 independent cultures). Cultures were treated with either scramble AUM (yellow); Fos AUM (magenta); Mapk1 and Mapk3 AUMs (orange); or Fos, Mapk1, and Mapk3 AUMs (blue). One-way ANOVA with Holm-Sidak post hoc test. *p ≤ 0.05; **p ≤ 0.01; ***p ≤ 0.001; ****p ≤ 0.0001. Data are represented as mean ± SEM.

To experimentally validate that p-ERK/p-FOS double-positive Th17 cells display heightened activation, we used flow cytometry to examine IL-2 and IL-17A production with respect to p-ERK1/2 and p-FOS staining. In a validation cohort of basal and PMA/ionomycin-stimulated cells processed with standard PFA/methanol fixation, we gated on p-ERK1/2^−^ p-FOS^+^, p-ERK1/2^+^ p-FOS^+^, and p-ERK1/2^+^ p-FOS^−^ cells (Figure 5A). In agreement with our inCITE-seq findings, p-ERK/p-FOS double-positive cells had the greatest IL-2 and IL-17A production among all three groups (Figure 5B). Irrespective of secondary stimulation, p-ERK/p-FOS double-positive cells consistently had the highest IL-2 and IL-17A MFI (Figure 5C) and percent positivity (Figure 5D). Moreover, a significantly increased percentage of p-ERK/p-FOS double-positive cells co-produced both IL-2 and IL-17A among the three phosphorylation groups irrespective of secondary stimulation (Figure 5E).

We next corroborated these findings by silencing either c-Fos and/or ERK1/2 signaling using antisense oligonucleotides from AUM Biotech, which bind to complementary target mRNA and block translation, thereby reducing protein levels. T cells polarized under Th17 conditions were treated with either scramble AUM or AUMs targeting c-Fos, ERK1/2, or a combination of all three (c-Fos and ERK1/2). Combination targeting of both c-Fos and ERK1/2 resulted in the greatest reduction in both the percentage and MFI of IL-2 and IL-17A (Figures 5F and 5G) compared to targeting c-Fos or ERK1/2 individually. We further validated these findings by measuring secreted protein concentrations of IL-2, IL-17A, IL-17F, tumor necrosis factor-α, and IL-10 in culture supernatants using a multiplex assay. Again, the greatest reduction in all these cytokines occurred with combinatorial targeting (Figure 5H).

### Early IL-2 production is associated with augmented Th17 cell maintenance and transdifferentitation

**Figure 6.**
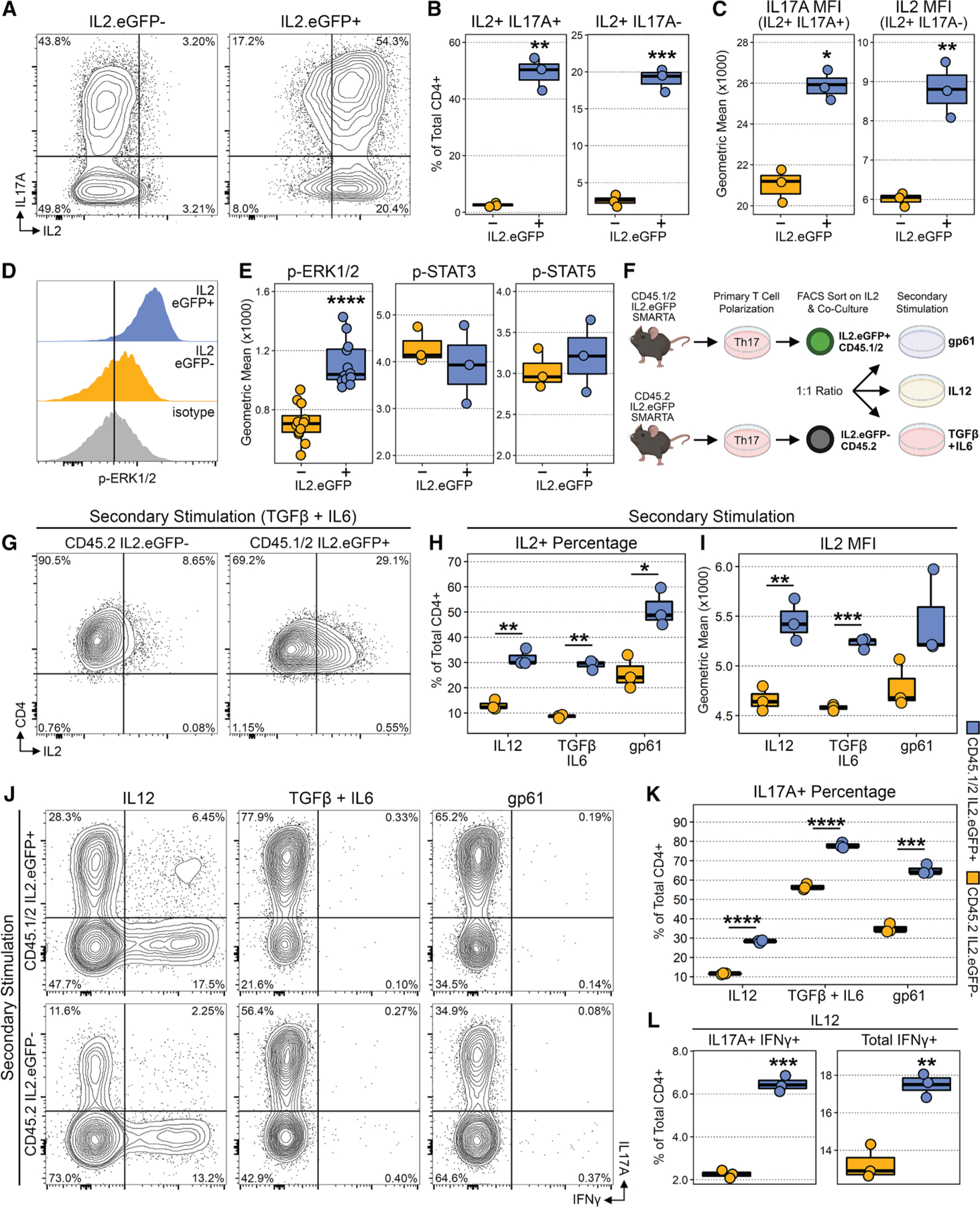
Early IL-2 production is associated with increased Th17 maintenance and transdifferentiation (A–E) Flow cytometry from Th17 polarized IL-2.eGFP-SMARTA T cells sorted at 40 h (*n* = 3 independent cultures) based on IL-2 (GFP) expression. (A) Flow cytometry plots showing expression of IL-17 and IL-2 in GFP^−^ (yellow) versus GFP^+^ (blue) cells. (B and C) Boxplots showing percentage (B) of IL2^+^IL17A^+^ and IL2^+^IL17A^−^ and MFI (C) of IL-17A and IL-2 in the FACS-sorted GFP^−^ (yellow) versus GFP^+^ (blue) populations. (D) Boxplots showing MFI of p-ERK1/2 and p-FOS staining based on IL-2 (GFP) expression. (E) Boxplots showing MFI of p-STAT3 and p-STAT5 staining based on IL-2 (GFP) expression. (F) Schematic of design for the SMARTA IL2.eGFP^+^CD45.1/45.2^+^ (green) and IL2.eGFP^−^CD45.2^+^ (black) co-culture experiment under different secondary stimulation conditions. TCR (gp61, purple), Th17 transdifferentiation (IL-12, yellow) and Th17 maintenance (TGF-β + IL-6, red). (G) Flow cytometry plots (*n* = 3 independent cultures) showing IL-2 expression in co-culture under Th17 maintenance conditions based on early IL-2 production. (H and I) Boxplots showing percentage positivity (H) and MFI (I) of IL-2 between SMARTA IL2.eGFP^−^CD45.2^+^ (yellow) and SMARTA IL2.eGFP^+^CD45.1/45.2^+^ (blue) in co-culture under Th17 transdifferentiation (IL-12), Th17 maintenance (TGF-β + IL-6), and TCR (gp61) conditions. (J) Flow cytometry plots (*n* = 3 independent cultures) showing IL-17 and IFN-γ production under different conditions outlined above during secondary stimulation. (K and L) Boxplots showing IL-17A (K), IL-17^+^/IFN-γ^+^ (L), and total IFN-γ^+^ (L) percent positivity under different co-culture conditions during secondary stimulation between SMARTA IL2.eGFP-CD45.2^+^ (yellow) and SMARTA IL2.eGFP^+^CD45.1/45.2^+^ (blue) cells. One-way ANOVA with Holm-Sidak post hoc test. *p ≤ 0.05; **p ≤ 0.01; ***p ≤ 0.001; ****p ≤ 0.0001. Data are represented as mean ± SEM.

IL-2 is produced early in T cell activation, and its production is linked to the strength of TCR and co-stimulatory signaling. Our group previously showed that naive T cells polarized under Th17 conditions produce high amounts of IL-2 relative to Th0, Th1, and Th2 conditions.^32^ However, it remains unclear whether IL-2 production is associated with any functional consequences in the Th17 pathway, including the maintenance of IL-17 expression or the ability of Th17 cells to transdifferentiate to IFN-γ producing Th1-like cells that are critical for inducing transfer colitis and experimental autoimmune encephalomyelitis (EAE) *in vivo*. In view of our Vivo-seq findings, which showed that p-ERK1/2 and p-FOS double-positive cells expressed a unique gene expression program linked to increased activation and cytokine production (Figure 4A), we next explored how hyperactivation affects the Th17 program by focusing on IL-2. Using the IL-2. eGFP-SMARTA TCR-transgenic mice described previously,^32^ we performed Th17 polarization and then sorted IL-2^+^ versus IL-2^–^ cells. IL-2^+^ cells had increased expression of IL-17 both by percentage of positive cells and MFI (Figures 6A–6C) as well as activation markers (CD44 and PD-1) (Figure S7A). Correspondingly, IL-2^+^ cells also had increased p-ERK1/2 and p-FOS signaling (Figure 6D), but no significant difference was observed between pSTAT3 (Y705) or pSTAT5 (Y694) levels (Figure 6E), which was in agreement with our previous findings between stimulated and unstimulated cells (Figure 2D). Transcriptionally, IL-2^+^ cells were significantly enriched for expression of *Il17a*, *Cd69*, *Tnfa*, *Cd40L*, *Jund*, and *Bcl6*, while IL-2^–^ cells had increased expression of *Prdm1* and *Cd25*, which was in alignment with our scRNA-seq data (Figures S7B and S8). Importantly, the reciprocal expression of *Bcl6* and *Prdm1* in IL-2^+^ and IL-2^–^ cells, respectively, was concordant with our previous findings in the context of a type I response against *Listeria monocytogenes*.^32^

To assess the functional implications of early IL-2 production on Th17 cell fate and phenotype, we next performed competitive co-cultures of IL-2^+^ versus IL-2^–^ cells using CD45.1/45.2^+^ and CD45.2^+^ IL-2eGFP-SMARTA TCR-transgenic mice (Figure 6F). After initial Th17 polarization, CD45.1/45.2^+^IL-2^+^ and CD45.2^+^IL-2^−^ cells were sorted and re-stimulated in a 1:1 ratio under Th17 maintenance conditions (transforming growth factor β [TGF-β] + IL-6), Th1 transdifferentiation conditions (IL-12), or TCR alone (gp61 peptide). Notably, cells that produced IL-2 early in the primary response produced more IL-2 with re-stimulation under all three conditions (Figures 6G–6I). Importantly, early IL-2 production was associated with increased IL-17 production under both Th17 maintenance and TCR conditions and increased ability to transdifferentiate to IFN-γ-producing Th1-like cells following exposure to IL-12 (Figures 6J–6L). Finally, we expanded our findings *ex vivo* using the Th17 transfer colitis model described previously by our group.^33^ In this model, the transferred Th17 cells undergo *trans*-differentiation into IFN-γ-producing Th1-like cells, which are critical for disease induction. For these experiments, similar to our *in vitro* co-culture experiments, IL2.eGFP^+^CD45.1/45.2^+^ and IL2.eGFP^−^CD45.2^+^ naive T cells were polarized under Th17 differentiation conditions and sorted at 40 h based on IL-2 expression. These IL-2^+^ CD45.1/45.2^+^ and IL-2^−^ CD45.2^+^ cells were then co-transferred intraperitoneally into Rag1^−/−^ recipients. Colons were harvested on day 14 post-transfer, and the T cells were analyzed by flow cytometry. We found a significantly higher number and percentage of IL-2^+^ IL-CD45.1/45.2^+^ T cells in the colon at day 14 compared to IL-2^−^ CD45.2^+^ cells (Figures S9A–S9D). Additionally, IL-2^+^ cells effectively transdifferentiated, as evidenced by increased IFN-γ expression while retaining IL-2 expression (Figures S9E and S9F).

Our findings support IL-2 as a marker for T cell activation, which has downstream consequences on Th17 cell fate and cellular phenotype. Thus, using Vivo-seq, we have identified distinct transcriptional signatures derived solely from patterns in phospho-signaling. This included a discrete and experimentally validated cell state, based on combinatorial phosphorylation, associated with heightened activation and IL-2 production during Th17 cell differentiation. We link this hyperactivated state to Th17 cell maintenance and transdifferentiation that have important functional consequences, especially in the context of Th17-mediated inflammatory disorders. Collectively, these data provide a first-of-its-kind demonstration that droplet-based scRNA-seq can be reliably integrated with phospho-signaling using DES fixation. We establish that coupling this previously hidden layer of biological information with single-cell sequencing is instructive of functionally distinct cell activation states and sets the stage for future investigations *in vivo*.

## DISCUSSION

Single-cell sequencing presents an opportunity to deconstruct cellular networks across health and disease, but significant limitations remain. Most notably, existing methods prevent the coupling of transcriptomic data with intracellular phospho-signaling, resulting in a loss of key biological data that are key determinants of cellular programming for alternate phenotypes and differentiation fates. Here, we introduce Vivo-seq, an innovative experimental platform wherein preservation with a next-generation fixative retains cellular states and allows the parallel quantification of transcription and intracellular phosphorylation in single cells, leading to novel insights into Th17 biology. Importantly, we utilize only commercially available reagents, making this platform immediately accessible to the research community. We show that DES preserves multiple domains of biological information, including RNA, proteins, cellular morphology, and PTMs, and allows long-term preservation of biological specimens, which we anticipate will be useful for establishing biobanks of human tissues and other valuable samples. Moreover, we validate an optimized and flexible method for performing intracellular CITE-seq in DES-fixed cells. Leveraging this approach to study four phosphorylated intracellular proteins during Th17 cell development, we reveal that a subset of cells with concurrent phosphorylation of ERK1/2 and c-FOS have the highest expression of activation genes, such as *Il2*, *Il17a*, *Il17f*, *Cd69*, and *Cd40lg*, and the lowest expression of suppressive and resting markers, such as *Socs2*, *Maf*, and *Tcf7*. We experimentally validate that Th17 cells with simultaneous phosphorylation of ERK1/2 and c-FOS produce the greatest amount of IL-2 and IL-17A and that concurrent expression of both ERK1/2 and c-FOS is required for maximum cytokine production. Finally, using IL-2 as a proxy to identify and isolate this hyperactivated subpopulation, we establish downstream functional consequences for Th17 cell maintenance and transdifferentiation with important implications for Th17-driven inflammatory disorders such as inflammatory bowel disease and EAE. We anticipate that Vivo-seq will broadly improve the study of signaling networks by preserving cellular states and allowing the parallel integration of transcription and intracellular signaling.

Coordinate signaling by the TCR, co-stimulatory receptors and cytokine receptors is deterministic for differentiating distinct CD4 T helper cell subsets. However, the current understanding of how these three priming signals and their respective intracellular signaling are integrated to shape cellular phenotypes is fragmented due to the limitations of reductionist approaches that focus on individual pathways.^34^ While these approaches are necessary to understand the discrete components of signaling networks, the generalizability of such results is challenged by the nature of intracellular signaling, which is context dependent (i.e., the signaling machinery present within a given cell), cooperative in nature (i.e., the interactions between discrete signaling cascades), and highly modular (i.e., the recombining of a fixed set of signaling modalities). We and others have previously shown that during T cell activation, a minimum threshold of TCR signal intensity is necessary for IL-2 production.^32,35,36^ The *Il2* promoter contains composite elements for binding of several transcription factors such as nuclear factor of activated T cells (NFAT), activator protein 1 (AP-1), nuclear factor κB (NF-κB), octamer-binding protein 1, and forkhead box protein P3 (FOXP3).^37,38^ Upon TCR stimulation and co-stimulation, ERK1/2 is activated via the MAPK (mitogen-activated protein kinase) pathway. Activated (phosphorylated) ERK1/2 (p-ERK1/2) translocates to the nucleus, where it regulates transcription factors, including AP-1 (c-FOS), NFAT, and NF-κB, which collectively drive potent Il2 transcription.^39–42^ Emerging evidence suggests that selective cooperation of these transcription factors at different gene loci can mediate distinct functions depending on the cellular context and the microenvironment. For instance, following antigenic stimulation, NFAT can induce *Il2* transcription by forming complexes with AP-1.^43^ Alternatively, FOXP3 can sequester NFAT in regulatory T cells to repress *Il2* and upregulate CD25 and CTLA4, thereby conferring suppressive effects.^44^ Vivo-seq herein has the potential to transform our understanding of how upstream signaling cascades influence intracellular signaling and consequent downstream gene targets by illuminating cooperativity among pathways at single-cell resolution and in a massively parallel fashion. Our data reveal that potentiation of cytokine production correlated with phosphorylation of ERK1/2 and c-FOS, phosphokinases downstream of TCR, and co-stimulation (signal 1 and 2), rather than STAT3 or STAT5, which are activated by cytokine signaling (signal 3). These findings are consistent with different thresholds and functions for each of the three T cell activation signals, supporting a model wherein TCR and co-stimulation activate downstream cascades that remodel genomic targets, thereby enhancing accessibility to cytokine-activated signals, such as STATs.

An intriguing finding that warrants further exploration is the association between T cell activation, cytokine production, and cellular fates. Our data show that early IL-2-producing Th17 cells continue to produce the greatest IL-2 and IL-17 with subsequent stimulations, are more predisposed to transdifferentiation to Th1-like cells, and sustain elevated expression of activation markers such as CD44 and PD-1. Conversely, IL-2 non-producers have increased expression of CD25, the high-affinity component of the IL-2 receptor, indicating that IL-2 non-producers have a heightened ability to sense and respond to paracrine IL-2 signaling, which has previously been linked to enhanced effector-like functions.^32,45^ These findings extend a previous study from our group showing that, in the context of a Th1 response, IL-2 producers are preferentially fated for T follicular helper (Tfh) development and central memory while IL-2 non-producers are preferentially fated for non-Tfh effector development and effector memory.^32^ They further suggest that early bifurcation of T cell fate driven by distinct phosphorylated signaling cascades engaged by differing strengths of TCR and co-stimulatory signaling may be generalizable to each of the T effector lineages and may be reinforced by divergent production and responsiveness to IL-2. Future studies will be needed to extend these findings *in vivo* and further explore the implications for alternative T cell programming afforded by coordinate analysis of proximal signaling and downstream gene expression networks.

In summary, we demonstrate the feasibility of performing intracellular CITE-seq in DES-fixed cells to integrate scRNA-seq and phospho-signaling. We identify a hyperactivated Th17 cell state characterized by dual expression of p-ERK1/2 and p-FOS associated with the highest IL-2 and IL-17 expression. We also unravel a functional consequence of early IL-2 production by developing Th17 cells with downstream effects on their plasticity. We anticipate that this platform will enable accurate reconstruction of cellular networks and uncover how cooperativity in signaling networks shapes cell phenotypes during homeostasis and pathophysiological states.

## RESOURCE AVAILABILITY

### Lead contact

Further information and requests should be directed to the lead contact, Robert S. Welner (rwelner@uab.edu).

### Materials availability

This study did not generate new unique reagents.

### Data and code availability

- Datasets related to this paper can be found at GEO: GSE297075.

## STAR★METHODS

### EXPERIMENTAL MODEL AND STUDY PARTICIPANT DETAILS

#### Study design

The objective of this study was to develop an improved cellular fixation strategy for the simultaneous quantification of RNA and intracellular phosphorylated proteins in single-cell sequencing. A secondary open-ended objective was to then apply this platform to Th17 development to identify and then experimentally validate a signaling network associated with heightened cytokine production. Sample sizes were based on resource availability and empirical effect sizes noted in preliminary experiments. Replicate sample sizes are noted in the figure legends, and no outlier samples were excluded. Male and female mice were used without randomization or blinding given the objective nature of our readouts such as flow cytometry and sequencing.

#### Mice

Wild-type (WT) mice (8–12 weeks, males, C57BL/6J) were purchased from Jackson Laboratory (Bar Harbor, ME). Il17a-eGFP mice and IL2.eGFP-SMARTA mice were described previously.^32,46^ All mice were housed at the University of Alabama at Birmingham (UAB). Mice were provided with food and water *ad libitum* and housed under a 12-h light-dark cycle. All experimental procedures were approved by the UAB Institutional Animal Care and Use Committee (IACUC).

#### Th17 cell culture and stimulation

Naive CD4 T cells were isolated as previously described.^15^ Briefly, mice were sacrificed, and spleens were dissected and mashed over a 70μm filter in complete IMDM media (IMDM media containing 10% FBS, 100 IU/mL penicillin, 100 μg/mL streptomycin, 1mM sodium pyruvate, 1x non-essential amino acids, 50μM β-mercaptoethanol, 1mM HEPES buffer, and 2mM glutamine). Red blood cells were removed using ACK lysis buffer, and naive CD4 T cells were purified using the MACS Miltenyi negative CD4 T cell selection kit (Militenyi; 130–104-454). To generate Th17 cells, naive CD4 T cells were cultured for 72h at 37°C in complete IMDM media (ThermoFisher; 12440046) containing plate-bound anti-CD3 (10μg/ml; clone 145–2C11), anti-CD28 (1μg/ml; clone 37.51), IL-6 (R&D Systems; 406-ML-025/CF; 20ng/ml), recombinant human TGF-β (R&D Systems; 240-B-010/CF; 2.5ng/ml), neutralizing anti-IFN-γ (10μg/ml; clone XMG1.2), and neutralizing anti-IL-4 (10μg/ml; clone 11B11). After 72h, Th17 cells were restimulated with phorbol myristate acetate (PMA; Millipore Sigma; P8139–1MG; 50ng/ml), ionomycin (Millipore Sigma; I3909–1ML; 750ng/ml) and BD GolgiStop (BD Biosciences; 554715) for 3 h at 37°C.

#### Adoptive model of Th17 transfer colitis

Naive T cells from IL2.eGFP^+^CD45.1/45.2^+^ and IL2.eGFP^−^CD45.2^+^ mice were polarized under Th17 conditions (as described before). At 40 h, live CD4^+^ T cells were FACS sorted based on IL-2 eGFP expression. IL-2^+^ (CD45.1/45.2^+^) and IL-2^−^ (CD45.2^+^) cells were co-transferred intraperitoneally (2 × 10^5^ each) into Rag1^−/−^ mice. Colons were harvested on day 14 for T cell analysis by flow cytometry.

### METHOD DETAILS

#### DES fixation of Th17 cells

DES fixation was performed using vivoPHIX (Rapid Labs Ltd., UK; RD-VIVO-50). For Th17 T cell fixation, culture media containing cells was transferred to 5mL FACS tubes and centrifuged at 4°C for 5 min at 300G. The supernatant was quickly decanted, and the cell pellet was loosened with light vortexing. ∼300μL of DES was added directly onto the cells in FACS tubes, followed immediately by vortexing to mix the cells into the DES. The DES-Th17 mixture was then incubated at room temperature for 2 h, after which aliquots were moved to 1.5mL microcentrifuge tubes and placed at −80°C for long-term storage.

#### Transfer from DES to aqueous buffer

Transfer of fixed cells from DES to aqueous buffer occurred following treatment with 10% glacial acetic acid (Fisher Scientific; A38S-500; AcOH), which we found was necessary to prevent RNase reactivation (Figure S1). If the samples were stored at −20°C or −80°C, the specimens were first allowed to acclimate to room temperature for 10–15 min. First, dissociated samples/cells were transferred to 5mL FACS tubes, and the volume of each sample was recorded. An equal volume of 20% AcOH/80% DES was then premixed, using vigorous vortexing to ensure complete mixing. Equal volumes of the 20% AcOH/80% DES mixture were then added to the FACS tubes containing the DES-fixed samples, and the tubes were vortexed at max speed for ∼10–15 s to ensure complete mixing. The samples were then incubated at room temperature for 5 min, and then ∼4mL of ice-cold rehydration buffer (3X saline sodium citrate (SSC; Invitrogen; AM9770) containing 8mM EDTA) was added to the samples. The FACS tubes were capped and inverted several times to ensure the complete dissolving of DES. The samples were then filtered through 40–70μm strainers into new FACS tubes, which were immediately centrifuged at 300G for 5 min in a centrifuge prechilled to 4°C. Following centrifugation, the supernatant was decanted, and any remaining large beads of solution along the inner walls of the FACS tubes were removed. The samples were then resuspended in staining buffer.

#### Blocking and immunostaining DES fixed cells

DES fixed samples were blocked in staining buffer (∼200–500μL depending on the experiment) and incubated on ice for ∼15–30 min to allow the blocking of non-specific epitopes. Staining buffer contained 1% BSA, 100U/mL heparin sodium salt (Millipore Sigma; H3393–100KU), 4mM EDTA, 5% True-Stain Monocyte Blocker, 5% TruStain FcX PLUS, 5% TruStain FcX, and 1x RNasin Plus (Promega; N2615) in 1x SSC. Following blocking, primary antibodies diluted in staining buffer were added to the FACS tubes, and samples were stained on ice for 30 min. Following staining, samples were diluted with 4mL of ice-cold wash buffer (1% BSA, 4mM EDTA in 1x SSC) and centrifuged at 300G for 5 min at 4°C to remove unbound antibodies. For flow cytometry, samples were resuspended in wash buffer, whereas for scRNA-seq, samples were resuspended in ice-cold 1x SSC containing 1x RNasin Plus and 0.04% BSA. Note that for phospho-staining, 1x Halt Phosphatase Inhibitor Cocktail (ThermoFisher; 78426) was added to the rehydration buffer, and 2x Halt Phosphatase Inhibitor Cocktail was added to the staining buffer. Lastly, for inCITE-seq, in addition to 2x Halt Phosphatase Inhibitor Cocktail, 1mg/mL of a 30-mer blocking oligonucleotide was added to the staining buffer. The 30-mer oligonucleotide (5′-CTAGACTGATTACGTACGTAAGATCGCTAC-3′) was designed to lack complementarity to any known endogenous mouse or human DNA sequences and contained a dideoxycytosine at the 3′ end to prevent extension by polymerases.

#### DES fixation of mouse bone marrow and colon

For mouse bone marrow fixation, a clean transverse cut was made using a razor blade in the mid-diaphysis of the femur shaft. The bone marrow was then centrifuged directly into DES, similar to a recent report describing the preservation of bone marrow with RNA-later.^47^ Specifically, the very end of a 0.6mL microcentrifuge tube was cutoff using a razor blade and placed inside a 1.5mL microcentrifuge tube that contained ∼100μL of DES. One-half of the femur was then placed in the 0.6mL tube, with the exposed bone marrow pointing downward toward the DES (Figure S1). The two stacked tubes were then centrifuged at 5,700G for 30 s. The upper chamber containing the 0.6mL tube and the empty femur shaft was removed, and an additional ∼200μL of DES was added on top. A clean 0.1–10μL pipette tip was then used to stir the bone marrow into the DES. The bone marrow-DES mixture was then incubated at room temperature for 2 h before storage at −80°C.

For mouse colon fixation, the transverse colon was acutely dissected, and feces were removed by washing the luminal contents out with 1x phosphate buffered saline (PBS). The lumen was then filleted open with a single cut and a ∼10mm long portion was obtained. Excess water was gently wicked away, and the colon was then submerged in ∼500μL of DES. After 2–3 h at room temperature, the DES-colon mixture was moved to 4°C for short-term storage prior to dissociation.

#### Dissociation of DES-Fixed mouse colon

Dissociation was performed in a 28/40khz dual-frequency ultrasonication water bath (Vevor, Walmart; UPC 650971639459). DES-fixed mouse colon were removed from the DES, padded dry using a Kimwipe, and then minced into small (∼1mm) pieces in ∼100μL of fresh DES using fine dissecting scissors. For bone marrow, any remaining large bone marrow plugs were minced directly in the original DES using fine dissecting scissors. The DES was then diluted to 90% DES/10% H_2_O by premixing DES with nuclease-free water containing 8mM EDTA and then transferring the minced tissue directly into it. The final volume of the tissue plus diluted DES was ∼200–300μL. The mixture was transferred to 1mL thick-wall polycarbonate ultracentrifuge tubes (Beckman Coulter; 355657), and the open ends were covered using pieces of transparent adhesive film. The tissue plus diluted DES was vortexed at max speed for ∼15–30 s to ensure complete mixing before being suspended in the ultrasonication water bath, which was prechilled to 4°C using ice. The samples were sonicated for 10 min, with pauses every 2–3 min for a brief (∼10 s) vortexing at max speed. Dissociation can be monitored visually through increases in turbidity. Typically, some small clumps of undissociated tissue will remain after 10 min of sonication and will be filtered out upon transfer to the aqueous buffer.

#### Bone marrow RNA electrophoresis

We optimized the transition from DES to aqueous buffer using RNA electrophoresis on DES-fixed mouse bone marrow samples (Figure S1). We tested three aqueous transfer strategies: (i) treatment with 10% AcOH followed by excess 3x SSC, (ii) excess 3x SSC only, as previously reported for methanol-fixed cells,^3^ and (iii) methanol treatment followed by resuspension in a high-salt buffer akin to diluted RNAlater (4M ammonium sulfate), as recently reported for methanol-fixed cells.^9,10^ The same bone marrow DES samples were split 3 ways to test each of the 3 methods in parallel. For the AcOH method, samples were treated with 10% AcOH for 5 min at room temperature, followed by washing with 4mL of ice-cold 3x SSC containing 8mM EDTA, as described above. After centrifugation, the solution was decanted, the cell pellet was loosened with light vortexing, and cells were lysed using Qiagen Buffer RLT, according to the manufacturer’s instructions. For the 3x SSX only method, the protocol was identical to that described using the AcOH method but without the 10% AcOH treatment step. For the methanol/high-salt buffer method, we adapted a recently described protocol used in methanol fixed cells.^9,10^ Briefly, in a 1.5mL microcentrifuge tube containing DES-dissociated bone marrow, 4 volumes of ice-cold methanol were added dropwise while gently vortexing the sample to ensure mixing. The mixture was then centrifuged at 300G for 3 min at 4°C. The methanol was removed and 500μL of ice-cold 3x SSC was gently added without disturbing the cell pellet. The 3x SSC was removed, and the cells were resuspended in 100μL of ice-cold high-salt buffer (3.4M ammonium sulfate, 50mM EDTA, 1x RNasin Plus in nuclease-free water, pH 5.2) and incubated on ice for 10 min. The cell mixture in high-salt buffer was then transferred to a FACS tube, 3mL of ice-cold 3x SSC was added, and the sample was centrifuged at 300G for 5 min at 4°C. After centrifugation, the solution was decanted, the cell pellet was loosened with light vortexing, and cells were lysed using Qiagen Buffer RLT, according to the manufacturer’s instructions. RNA purification and DNase treatment were performed using the Qiagen RNeasy Plus Mini kit (Qiagen; 74134) according to the manufacturer’s instructions. The quality of purified RNA was then visualized with electrophoresis using a 1% bleach gel, prestained with ethidium bromide, as previously described.^48^

#### RT-qPCR and RNA integrity in Th17 T cells

Th17 T cells were differentiated in culture for 3 days as described. Th17 cells used for RT-qPCR and RNA integrity number analysis experiments did not receive secondary stimulation with PMA/ionomycin. DES fixation was performed as described above, and aliquots of unfixed viable cells were used for comparison. After 2 h of DES fixation at room temperature, aliquots of DES fixed cultures were stored at −20°C or −80°C for 1 month. DES fixed cells were treated with 10% AcOH and transferred to aqueous buffer, as described above. DES fixed cells were stained with 0.01mM SYTO40 in staining buffer on ice for 15 min. Viable cells were stained with viability dye on ice for 15 min 100,000 DNA+ cells (or 100,000 live cells for viable samples) were then FACS sorted directly into 500 μL of Trizol per sample. RNA purification and DNase treatment were performed using the Direct-zol Miniprep kit (Zymo Research; R2051) according to the manufacturer’s instructions. cDNA was generated using the Superscript IV VILO master mix kit according to the manufacturer’s instructions. qPCR was performed in 20μL reactions using SsoAdvanced Universal SYBR Green Supermix (Bio-Rad; 1725270) and a Bio-Rad CFX96 thermocycler. Raw Ct values were used for analysis. RIN analysis was performed using an Agilent 2100 per the manufacturer’s instructions.

#### Th17 flow cytometry

All flow cytometry data were analyzed using FlowJo (Version 10.8) and the details of all antibodies used in the study are provided in Key resources table.

#### Il17a.eGFP experiments

Flow cytometry was performed on Th17 T cells for IL17A protein and GFP from Il17a.eGFP mice. After 3 days of differentiation, Th17 cell cultures (*n* = 3) were split and half were given a secondary stimulation with PMA/ionomycin and BD GolgiStop for 3 h, as described above. These control and stimulated cultures were then split a final time, with half using DES for fixation/permeabilization and the other half with PFA/methanol using the BD Cytofix/Cytoperm Kit. Supernatant was discarded and cell pellets were loosened with light vortexing before adding either ∼300μL of DES or ∼300μL of ice-cold Cytofix/Cytoperm. DES samples were fixed for 2 h at room temperature while PFA/methanol samples were fixed for 30 min on ice before being exchanged with BD Perm/Wash Buffer. After 2 h of fixation, DES samples were treated with AcOH, moved through 3x SSC to 1x SSC, and incubated in staining buffer for 15 min, as described in detail above. Both DES and PFA/methanol cells were then stained with APC anti-IL17A (1:100) and unconjugated chicken anti-GFP (1:200) for 30 min on ice. Cells were washed with their respective wash buffers and then stained with Alexa Fluor Plus 488 anti-chicken secondary antibody (1:750) and SYTO40 (ThermoFisher; S11351; 0.01mM) for 30 min on ice. Samples were washed a final time and then resuspended in either FACS buffer for PFA/methanol or 1% BSA, 4mM EDTA in 1x SSC for DES cells. For both fixation types, APC conjugated rat isotype controls were included as well as controls without primary antibody for the AF+488 secondary stain (Figure S2). Samples were quantified using a BD FACSymphony A5.

#### Phosphorylation panel

Flow cytometry for phospho-epitopes was performed on DES-fixed Th17 T cells (*n* = 3 cultures) that were either left in basal culture medium (unstimulated) or treated with PMA/ionomycin for 3 h (stimulated), as described previously. After 2 h of fixation in DES at room temperature, samples were stored at −80°C for several days. Samples were allowed to warm to room temperature for ∼15 min after which aliquots of each were moved to FACS tubes. Samples were treated with AcOH and moved through 3x SSC to staining buffer, as described previously but with the addition of 1x Halt Phosphatase Inhibitor Cocktail in the rehydration buffer and 2x Halt Phosphatase Inhibitor Cocktail in the staining buffer. After blocking for 15 min, samples from each culture and condition were then counted and diluted to the same concentration of cells/μL. Samples were then split 15 ways in equal volumes and stained with 1 of 13 different unconjugated anti-phospho-epitope antibodies (Figure 2D). Isotype controls for both rabbit and mouse were included. Cells were washed with wash buffer (1% BSA, 4mM EDTA in 1x SSC) and then stained with Alexa Fluor Plus 647 anti-rabbit or anti-mouse secondary antibodies (1:750) and SYTO40 (0.01mM) for 30 min on ice. Samples were washed one final time and then resuspended in wash buffer. In addition, an aliquot of each culture was stained with PE conjugated anti-RORγt and corresponding isotype controls were included (Figure S2). Samples were quantified using a BD FACSymphony A5.

#### c-FOS/ERK validation experiments

Flow cytometry for IL-2 and IL-17A was performed on PFA/methanol fixed Th17 T cells (*n* = 5 cultures) that were either left in basal media or treated with PMA/ionomycin for 3 h, as described previously. After 3 h, cultures were moved to FACS tubes and centrifuged at 4°C in undiluted culture medium (300G for 5 min). Supernatant was discarded and cell pellets were loosened with light vortexing before adding ∼300μL of ice-cold Cytofix/Cytoperm. Samples were incubated on ice for 1 h before being exchanged with BD Perm/Wash Buffer containing 1x Halt Phosphatase Inhibitor Cocktail and 0.1% Triton X-100 for nuclear permeabilization. After 30 min of permeabilization on ice, samples were exchanged with BD Perm/Wash Buffer containing 1x Halt Phosphatase Inhibitor Cocktail and then stained with unconjugated rabbit anti-*p*-ERK1/2 (1:33 dilution) for 30 min on ice. After washing off the staining buffer, samples were resuspended in BD Perm/Wash Buffer containing 1x Halt Phosphatase Inhibitor Cocktail and anti-rabbit Alexa Fluor Plus 488 secondary antibody (1:500 dilution) for 20 min on ice. After washing off the secondary antibody, samples were again resuspended in BD Perm/Wash Buffer containing 1x Halt Phosphatase Inhibitor Cocktail and then stained with PE-conjugated anti-IL2 (1:100 dilution), AF647-conjugated *p*-FOS (1:50 dilution), and SYTO40 (0.01mM) for 30 min on ice. Samples were washed a final time and then resuspended in FACS buffer and quantified using a BD FACSymphony A5. AF647-conjugated rabbit isotype, unconjugated rabbit isotype, and PE-conjugated rat isotype controls were included.

#### IL-2.eGFP SMARTA experiments

Naive SMARTA Tg T cells were isolated from the spleens of IL-2.eGFP SMARTA CD45.1/45.2 and 45.2 mice as above and stimulated for 40 h with irradiated CD4-depleted feeders and the LCMV gp61 peptide (2.5 μg/mL) recognized by the SMARTA TCR under Th17 polarizing conditions as described above. At 40 h, FACS purified live CD4^+^ CD45.1/45.2^+^ IL-2^+^ (GFP+) and live CD4^+^ CD45.2^+^ IL-2^−^ (GFP-) cells were co-cultured at 1:1 ratio with CD4-depleted feeders and re-stimulated for 48 h under Th17 maintenance conditions (as above), Th17 transdifferentiation conditions (with IL-12 at 10 ng/mL, neutralizing anti-IFN-γ at 10μg/ml, and neutralizing anti-IL-4 at 10μg/ml) or TCR alone (gp61 at 2.5 μg/mL, neutralizing anti-IFN-γ at 10μg/ml, and neutralizing anti-IL-4 at 10μg/ml).For the IL-2. eGFP SMARTA CD45.1/45.2 and 45.2 co-culture experiment, cells were taken following 48 h of restimulation under Th17 maintenance, Th17 transdifferentiation and TCR conditions and then stimulated with PMA/Ionomycin and BD GolgiStop for 3 h. Cells were then stained for surface and intracellular markers using BD Cytofix/cytoperm as described previously.

#### AUM gene silencing experiments

For silencing c-FOS and ERK1/2, we used AUM*silence* single-stranded antisense oligonucleotides (AUMs) from AUM Biotech, which do not require transfection reagents and degrade target mRNA through RNase H-mediated cleavage. For each specific mRNA target (Fos, Erk1, Erk2), 5 unique AUMs were designed, each against a different region of the mRNA target sequence. Sequence design was done by AUM Biotech. These lyophilized AUMs were then resuspended in nuclease-free water to a stock concentration of 100μM and each of the 5 unique AUMs per mRNA target were then mixed together in an equal molar ratio. For experiments, AUMs were diluted in culture media to a final concentration of 1μM per mRNA target and were added 1h prior to PMA/ionomycin stimulation on culture day 3. This time point was chosen to allow adequate Th17 differentiation and IL-17 production. For flow cytometry experiments, GolgiStop was added along with the PMA/ionomycin. For cytokine secretion experiments, culture supernatants were collected, cellular debris was removed via centrifugation, and secreted cytokines were quantified using the LEGENDplex Mouse T Helper Cytokine Panel bead array (BioLegend; 741044) per manufacturer’s instructions.

#### Imaging flow cytometry

Imaging flow cytometry was performed using an Amnis ImageStream MKII. Briefly, DES-fixed mouse colon was dissociated in DES as described above. Following dissociation, DES-fixed samples were transferred to FACS tubes and an equal volume of 8% PFA was added directly to the DES followed by gentle stirring with a pipette tip. Additional fixation was done to reinforce delicate structures that can be sheared upon transfer to aqueous buffer, especially when multiple rounds of centrifugation are required. The cells were allowed to fix on ice for 10 min in 4% PFA, after which 4mL of ice-cold 3x SSC was added. The cell suspensions were then filtered using 5μm track etched hydrophilic membranes (Millipore Sigma; WHA10417406) under light suction. The cells were then gently washed off of the membranes into FACS tubes by placing the membranes directly into ∼500μL of staining buffer. 0.01mM SYTO40 was then added to the FACS tubes, and the cells were stained for 15 min on ice. After nuclear staining, the samples were again washed and filtered as described above before being resuspended in ∼100μL of wash buffer. The cells were then loaded on the Amnis ImageStream and brightfield, side scatter, and DAPI channels were used for capturing images. IDEAS (version 6.2) software was used for quality control filtering, image optimization, and exporting captured images. Photoshop was used to crop selected images for creating the collages in Figure 1.

#### Confocal immunofluorescence

Confocal immunofluorescence was performed on PFA-fixed mouse colon. Large intestine was dissected from an 8-week-old WT mouse, flushed of luminal contents, and fixed overnight in 4% PFA at 4°C. Samples were then embedded in paraffin and 5μm thick sections were cut. For staining, the slides were first incubated for 2 h at 60°C. Deparaffinization and rehydration was performed with three sequential 5 min incubations in xylene, two sequential 5 min incubations in 100% ethanol, two sequential 5 min incubations in 95% ethanol, and washed in distilled water in three sequential 5 min incubations with gentle agitation. Citrate buffer prewarmed to 70°C was used for antigen retrieval and was incubated in a heated steamer for 20 min, followed by three washes in distilled water with gentle agitation. Sections were incubated with 1x PBS for 10 min and were then blocked with Trident Universal Protein Blocking Reagent (GeneTex; GTX30963) and 5% TruStain FcX PLUS at room temperature for 1 h. Slides were stained overnight at 4°C with a rabbit primary antibody to E-Cadherin (1:100) and fluorescein-conjugated UEA-1 Lectin (Vector Labs; FL-1061–2; 1:200). The slides were washed in excess 1x PBS and then incubated with anti-rabbit Alexa Fluor plus 647 in 1% BSA and 1% horse serum in 1x PBS at room temperature for 1 h. The slides were again washed in 1x PBS followed by Hoechst staining for 5 min at room temperature. After one final wash in 1x PBS, the slides were mounted with ProLong Gold Antifade mountant (ThermoFisher; P36930), pressed flat for 10 min, and then imaged on a Nikon AX confocal.

#### Immunoblotting for bone marrow post-translational modifications

As described in Figure 2A, we isolated femurs from individual mice (*n* = 5), cut the bones in half, and briefly centrifuged the bone marrow into two separate solutions: (i) lysis buffer, or (ii) DES. The same lysis buffer was used for both experimental groups (RIPA buffer with 2x Halt Protease and Phosphatase Inhibitor Cocktail). For the DES group, ∼200μL of additional DES was added on top following centrifugation. The cells were left as a thin layer and submerged in DES for 2 h at room temperature. After incubation, the thin layer of cells was removed from DES using forceps, padded dry with a kimwipe, and chopped into tiny fragments using a razorblade on a Petri dish containing ∼500μL of lysis buffer. All samples were allowed to incubate on ice for 1 h in lysis buffer with periodic vortexing. After 1 h, the samples were sonicated using a probe sonicator (3 × 10s pulses at 30% power on ice). The samples were then centrifuged at 10,000G for 10 min at 4°C, and the supernatant was collected and stored at −80°C. Protein was quantified using the Pierce BCA Protein Assay Kit (ThermoFisher; 23225). Protein was linearized in Laemmli buffer (Li-Cor; 928–40004) at 100°C for 10 min, and then 20μg per sample were loaded onto 18 well 10% Criterion TGX Stain-Free Protein Gels (Bio-Rad; 5678034). Following electrophoresis, total protein stains were activated using a Bio-Rad ChemiDoc Imaging System per the manufacturer’s instructions and then transferred onto 0.2μm nitrocellulose membranes using the Trans-Blot Turbo Transfer System (Bio-Rad; 1704271) per the manufacturer’s instructions. After transfer, images of total protein stains were obtained. The blots were then treated with Pierce SuperSignal Western Blot Enhancer (ThermoFisher; 21050) per the manufacturer’s instructions and then blocked for 1 h at room temperature using Intercept (TBS) Blocking Buffer (Li-Cor; 927–60001). After washing with 1x TBS (Bio-Rad; 1706435), blots were incubated overnight with gentle rocking at 4°C with the anti-phospho-threonine (1:1000) primary antibody diluted in primary antibody buffer from the Pierce SuperSignal Western Blot Enhancer kit. The next day, blots were washed several times with 0.1% Tween 20 in 1x TBS and incubated for 1 h with secondary antibodies diluted in Intercept T20 (TBS) Antibody Diluent. Alexa Fluor Plus 800 secondary antibodies were used and diluted at 1:5000. The blots were then washed several times with 0.1% Tween 20 in 1x TBS and then 2 washes in 1x TBS without Tween 20 before being imaged on a Bio-Rad ChemiDoc Imaging System.

#### Oligonucleotide-antibody conjugation for intracellular CITE-Sequencing

Custom oligonucleotide-antibody conjugates were fabricated for intracellular CITE-seq (inCITE-seq). Custom 80 base-pair (bp) oligonucleotides, based on a modified TotalSeq-B design, were produced by IDT. The oligos had a 34bp 5′ PCR handle followed by 7 random spacer nucleotides (designated with N’s): 5′- GTGACTGGAGTTCAGACGTGTGCTCTTCCGATCTNNNNNNN −3’. A 22bp 3′ capture sequence was used, which was preceded by 6 random spacer nucleotides: 5′- NNNNNNGCTTTAAGGCCGGTCC-TAGCAA −3’. The final 2 adenine bases at the 3′ end of the capture sequence used phosphorothioated bonds. Finally, between the 2 sets of random spacer nucleotides was an 11bp unique barcode. 3 of these 80bp custom oligonucleotides were obtained with their own unique 11bp barcodes (barcode1: GTCACTACGAG, barcode2: TGGCTACAAGT, and barcode3: CGACATTGACA) and were conjugated at the 5′ end to a proprietary Protein G derivative with inducible cross-linking moieties upon UV light activation (oYo-Link; AlphaThera; AT1002–25ss). This product results in high-efficiency and site-specific conjugation to the Fc region of a broad diversity of IgG isotypes from many different source species; 2 oYo-links bind per antibody molecule.

The procedure for conjugation of inCITE-seq antibodies was performed as described below. The following antibodies (Ab) were conjugated: p-STAT3 Y705 (3μg Ab; clone 4/P-STAT3; barcode1), *p*-ERK1/2 T202/Y204 (1.5μg Ab; clone 197G2; barcode1), p-p65 S536 (1μg Ab; clone 93H1; barcode2), *p*-FOS S32 (0.5μg Ab; clone D82C12; barcode3), and rabbit isotype (3μg Ab; clone DA1E; barcode3). The oligo-conjugated oYo-links were reconstituted to 33μM and added to the respective antibody aliquots at a molar ratio of 1 Ab: 5 oYo in clear 0.6mL microcentrifuge tubes. The Ab/oYo solution was gently pipetted up and down to mix and then incubated at room temperature for 10 min. After this, the Ab/oYo mixtures were placed on ice and exposed to UV light for 2 h using the LED PX2 Photo-Crosslinking Device (AlphaThera Inc.).

After cross-linking, excess unbound oYo-link was removed (Figure S3) using streptavidin-conjugated magnetic beads (ThermoFisher; 65601) that were preincubated with biotinylated human IgG Fc fragments (Jackson ImmunoResearch; 009–060-008). First, 560μL of beads (∼62.5μL of beads per μg of antibody) were washed 3x with 1mL of 1x PBS containing 0.01% Tween 20 (PBST). The beads were then resuspended in 560μL of PBST and 67μL of IgG Fc (resuspended in nuclease-free water at 8.35mg/mL) was added to give a final concentration of 1μg Fc per 1μL beads. The 1.5mL microcentrifuge tube containing the mixtures were then incubated on a vortexer at low-speed for 30 min at room temperature to allow binding. After incubation, the bead mixture was then washed 3x with PBST and then 2x with 1x PBS, before being resuspended in 40μL of 1x PBS. For each 1μg of Ab used in the oYo-link conjugation, 4 μL of bead mixture was added to the tube. The Ab/oYo-link/bead mixture was then incubated on a vortexer at low-speed for 1 h at room temperature. 3 sequential magnetic separations were then performed by incubating the mixture on a magnetic rack for 5 min at room temperature and then transferring the supernatant to a new tube and repeating.

Lastly, the cleaned-up Ab-oligonucleotide conjugates were then incubated with E. coli ssDNA binding protein (EcoSSB; Promega; M3011) to prevent off-target oligonucleotide interactions, as recently reported.^14^ To do this, we first calculated the number of moles of oYo-link added per antibody; this equated to ∼3.3 × 10^−11^ mole per μL of oYo-link used (applicable when oYo-link is resuspended to 33μM, per the manufacturer’s instructions). After calculating the number of moles of oYo-link used for each antibody, we then multiplied this number by 12 for the number of moles of EcoSSB to be used per Ab reaction, which was based on the notion that each tetramer of EcoSSB binds to ∼35bp of ssDNA.^14^ Based on the molecular weight of EcoSSB, this ultimately equated to adding ∼7.5μg of EcoSSB per 1 μL of oYo-link used. This calculation purposely allowed excess EcoSSB, ensuring complete saturation of oligonucleotides. For each oligonucleotide-Ab conjugate mixture, 5μL of 10x NEBuffer4 (NEB; B7004S), the required volume of EcoSSB, and nuclease-free water were combined to give a final total volume of 50μL. The mixtures were then incubated at 37°C for 30 min in a thermocycler to allow EcoSSB binding. No further purification was used.

### QUANTIFICATION AND STATISTICAL ANALYSIS

#### Th17 T cell single-cell RNA-Sequencing

The same stimulated and unstimulated cultures (*n* = 3) from the phospho-flow cytometry experiment described earlier were used for scRNA-seq. Samples were allowed to warm to room temperature for ∼15 min after which aliquots of each were moved to FACS tubes. Samples were then treated with 10% AcOH for 5 min at room temperature and then 4mL of ice-cold 3x SSC containing 8mM EDTA and 1x Halt Phosphatase Inhibitor Cocktail was added. Samples were filtered and centrifuged in a prechilled centrifuge at 300G for 5 min. The supernatant was decanted, and the samples were resuspended in 500μL of ice-cold staining buffer (1x SSC in nuclease-free water containing 1% BSA, 100U/mL heparin sodium salt, 4mM EDTA, 5% True-Stain Monocyte Blocker, 5% TruStain FcX PLUS, 1x RNasin Plus, 2x Halt Phosphatase Inhibitor Cocktail, and 1mg/mL of blocking oligonucleotide). Samples were allowed to block on ice for ∼30 min during which the cell concentration of each sample was manually counted. The samples were then diluted to an equal concentration of cells/μL (1.5 million cells per mL) using the same staining buffer as above. Each sample was then split into even volumes and inCITE-seq was performed using two separate panels of antibodies, thereby generating 12 total unique samples (*n* = 3 unstimulated cultures + *n* = 3 stimulated cultures x 2 panels of inCITE-seq antibodies). All samples were hashed into the same well for 10X Genomics 3′ V3 to control for any potential well-to-well variations in capture efficiency, sample handling, or sequencing. The following hashing scheme was used:

All unstimulated culture samples were stained with TotalSeq-B0301 anti-mouse Hashtag1 antibody (designated: Hashtag1) and all stimulated culture samples were stained with Hashtag2. In addition to these hashes, one of six different hashes was used to distinguish each culture and panel combination: cultures A, B, and C for panel1 were stained with Hashtags 3, 4, and 5, respectively while cultures A, B, and C for panel2 were stained with Hashtags 6, 7, and 8, respectively.

Panel1 for inCITE-seq was stained with p-STAT3 Y705 (barcode1), p-p65 S536 (barcode2), and rabbit isotype (barcode3). Panel2 was stained with *p*-ERK1/2 T202/Y204 (barcode1) and *p*-FOS S32 (barcode 3). After EcoSBB incubation, all antibodies were chilled on ice and antibodies for the aforementioned panels were combined. Staining buffer, as described above, was added to each panel to a final volume of 175μL and 0.02mM SYTO40 was added. Each panel was then split evenly into 2 parts (87.5μL each) and then 0.875μg (or 1.75μL) of either Hashtag1 or Hashtag 2 was added. Each panel/hashtag combination was then evenly divided 3 ways (28μL each) into clean, empty FACS tubes, for a total of 12. For each culture/panel combination, 0.6μL of the respective Hashtag was added to each tube. Finally, 175μL of each of the respective cultures was added to the designated tubes and the samples were stained on ice for 30 min. After 30 min, samples were washed 2x with 4mL of ice-cold wash buffer (1% BSA, 4mM EDTA in 1x SSC). Each sample was resuspended in 250μL of 1x SSC containing 0.04% BSA and 1x RNasin Plus. 10,000 DNA+ cells from each of the 12 tubes were then FACS-sorted into the same tube. The final cell concentration was counted, and cells were loaded into one 10X Genomics 3′ V3 well. The CITE-Seq library, which included the hashtag and inCITE-seq antibodies, was sequenced to a depth of roughly 36,000 reads per cell.

#### Analysis of single-cell RNA-Sequencing

CellRanger count was used for demultiplexing, genome alignment, and antibody UMI counting. scAR was used to remove ambient RNA.^49^ Hashing cutoffs were determined visually using histograms. Doublets were defined as cells that were above the positive staining cutoff for 2 or more culture/panel-specific hashes or above the positive staining cutoff for both stim/unstim-specific hashes. Doublets were removed, as were cells that did not have any positive staining for a culture/panel-specific hash and/or a stim/unstim-specific hash. Quality control filtering was performed using a cutoff of 500 for total genes and 500 for total counts per cell and a cutoff of 0.1 was used for mitochondrial transcript proportion per cell. Preliminary UMAP dimensionality reduction and Leiden clustering revealed a small population of contaminating B cells (∼7% of the total dataset), which were subsequently removed (Figure S4).

Lastly, we used scVI modeling^50^ to compute a latent space representation for UMAP dimensionality reduction. This model used 2,000 highly variable genes, 1 categorical covariate (the combined identifier for culture, panel, and condition [i.e., stimulated or unstimulated]), and 4 continuous covariates (natural log of total counts per cell, natural log of total genes per cell, and ‘‘G2M’’ and ‘‘S’’ scores). The resulting latent space was then transferred to the non-subsetted dataset, which was subsequently used to compute nearest neighbors followed by dimensionality reduction using the UMAP algorithm in Scanpy.^51^ Finally, SCRAN was used^52^ for cluster-wise size factor normalization of the denoised raw counts expression data. After dividing each expression value by the respective size factor, we then used natural log+1 transformation to complete normalization.

#### Analysis of intracellular CITE-Sequencing

For inCITE-seq, we calculated the median value of rabbit isotype counts per cell for each culture and stim/unstim combination (i.e., cultureA_stimulated, cultureA_unstimulated, etc; hereafter referred to as ‘‘samples’’). For each sample, we then subtracted the median isotype value from the raw counts of the 2 phospho inCITE-seq antibodies per cell. If the subtraction resulted in a negative count for an inCITE-seq antibody in a given cell (i.e., if the median isotype value was greater than the counts for an inCITE-seq antibody), then we clipped the resulting value at 0. Median isotype counts showed little variation across samples, ranging from 3 to 5. The isotype corrected inCITE-seq counts were then natural log+1 transformed to give the final values. We annotated cells as positive for a given inCITE-seq phospho-target if a cell had >0 counts after isotype correction and natural log+1 transformation.

#### Differential gene expression analyses

Differential gene expression (DGE) analyses were performed on natural log+1 transformed; size factor normalized expression data (SCRAN). The R package Seurat^53^ was used for DGE computation using the MAST^54^ implementation of the FindMarkers() function. The following parameters were used: ‘‘min.pct’’ was set to 0.01 to eliminate genes expressed in <1% of cells, ‘‘logfc.threshold’’ was set to 0 thereby not filtering genes based on fold change size, and ‘‘mean.fxn’’ was set to ‘‘rowMeans’’ thereby using group mean expression for Log fold-change calculations. In addition, for the bone marrow scRNA-seq ‘‘latent.vars’’ was set to natural log genes per cell and the sample identifier. For the Th17 inCITE-seq, ‘‘latent.vars’’ was set to natural log genes per cell.

#### Intersection analyses of differentially expressed genes

Output files from FindMarkers() were used for analyses of shared differentially expressed genes (DGs) between groups (phosphor-targets in Figure 4). Gene names and direction of change for each group were combined for both intersection analyses. The R package ggupset was used for visualization.

#### Statistical analysis

Statistical analyses were performed using the R package rstatix or GraphPad Prism (version 6.01). Sample sizes and statistical tests are listed in the figure legends. All graphs with error bars report mean ± SEM values. Data were visualized using the R package ggplot2 with the exception of UMAP plots, which were generated using Scanpy. Correction for multiple hypothesis testing was applied where indicated. Detailed statistical information for each experiment, including the specific statistical tests employed, sample sizes, and significance levels, are provided in the corresponding figure legends and within the Results section where applicable. Sample size (n) represents the number of independent biological replicates, which may be individual animals, separate cell culture preparations, or independent experimental units, as specifically defined for each dataset in the corresponding figure legends.

## Supplementary Material

1

Supplemental information can be found online at https://doi.org/10.1016/j.celrep.2025.116006.

## Figures and Tables

**Table:** KEY RESOURCES TABLE

REAGENT or RESOURCE	SOURCE	IDENTIFIER
Antibodies		

PE anti-CD45 (30F11)	BioLegend	103105
PE anti-CD45R (B220)	BioLegend	103207
APC anti-Ter119 (Ter-119)	BioLegend	116211
AF647 anti-Spectrinβ (B-1)	Santa Cruz	sc-374309 AF647
APC anti-IL17A (eBio17B7)	ThermoFisher	25–7177-81
PE/Cy7 anti-IL17A (eBio17B7)	ThermoFisher	25–7177-82
PE anti-IL2 (JES6–5H4)	BioLegend	503807
PE anti-RORγT (AFKJS-9)	ThermoFisher	12–6988-82
anti-phos-STAT1 Y701 (D4A7)	Cell Signaling	7649
anti-phos-STAT1 S727 (D3B7)	Cell Signaling	8826
anti-phos-STAT3 Y705 (4P/STAT3)	BD Biosciences	612357
anti-phos-STAT3 S727 (D4X3C)	Cell Signaling	34911
anti-phos-STAT5 Y694 (D47E7)	Cell Signaling	4322
anti-phos-AKT T308 (C31E5E)	Cell Signaling	2965
anti-phos-AKT S473 (D9E)	Cell Signaling	4060
anti-phos-ERK1/2 T202/Y204 (D13.14.4E)	Cell Signaling	4370
anti-phos-ERK1/2 T202/Y204 (197G2)	Cell Signaling	4377
anti-phos-MEK1/2 S221 (166F8)	Cell Signaling	2338
anti-phos-CREB S133 (87G3)	Cell Signaling	9198
anti-phos-FOS S32 (D82C12)	Cell Signaling	5348
anti-phos-JUNB T102/T104 (D3C6)	Cell Signaling	8053
anti-phos-p65 S536 (93H1)	Cell Signaling	3033
AF647 anti-phos-FOS S32 (D82C12)	Cell Signaling	8677
anti-GFP (polyclonal)	Abcam	ab13970
BV510 anti-CD44 (IM7)	Biolegend	103044
PE-Cy7 anti-PD-1 (J43)	eBioScience	25–9985-82
APC anti-CD-25 (PC 61)	Biolegend	102012
e450 anti-CD-69 (H1.2F3)	Invitrogen	48–0691-80
PerCP-Cy5.5 anti-IL17A (TC11–18H10)	BD Biosciences	560666
e450 anti-IFNγ (XMG 1.2)	Invitrogen	48–7311-82
BV510 anti-CD-4 (RM4–5)	Biolegend	100553
PE anti-phos-STAT3 Y705 (13A3–1)	Biolegend	651004
AF647 anti-phos-STAT5 Y694 (47/Stat5)	BD Biosciences	612599
AF647 anti-phos-ERK1/2 pT202/pY204 (20A)	BD Biosciences	612593
anti-Phospho Threonine (42H4)	Cell Signaling	9386
anti-E-cadherin (24E10)	Cell Signaling	3195
AF647 anti-rhodopsin (RET-P1)	Santa Cruz	sc-57433 AF647
AF647 anti-Iba1 (EPR16588)	Abcam	ab225261
FITC anti-CD45 (30F11)	BioLegend	103107
Biotinylated anti-LEPR (polyclonal)	R&D Systems	BAF497
AF+488 anti-rabbit (polyclonal)	ThermoFisher	A32790
AF+647 anti-rabbit (polyclonal)	ThermoFisher	A32795
AF+488 anti-chicken (polyclonal)	ThermoFisher	A32931
AF+800 anti-rabbit (polyclonal)	ThermoFisher	A32808
AF+800 anti-mouse (polyclonal)	ThermoFisher	A32789
APC rat isotype (G155–178)	BD Biosciences	550882
PE rat isotype (MPC-11)	BioLegend	400312
AF647 rabbit isotype (DA1E)	Cell Signaling	2985
Rabbit isotype (DA1E)	Cell Signaling	3900
Mouse isotype (G3A1)	Cell Signaling	5415
True-Stain Monocyte Blocker^™^	BioLegend	426102
Mouse TruStain FcX^™^ anti-CD16/32 (93)	BioLegend	101319
Mouse TruStain FcX^™^ PLUS anti-CD16/32 (S17011E)	BioLegend	156603
Human TruStain FcX^™^	BioLegend	422301
Neutralizing anti-IL4 (11B11)	BioLegend	504121
Neutralizing anti-IFNγ (XMG1.2)	BioLegend	505833
anti-CD28 (37.51)	BioLegend	102115
anti-CD3 (145–2C11)	ThermoFisher	16–0031-82
LCMV gp61	Anaspec	AS-64560
TotalSeq^™^-B0301 anti-mouse Hashtag 1	BioLegend	155831
TotalSeq^™^-B0302 anti-mouse Hashtag 2	BioLegend	155833
TotalSeq^™^-B0303 anti-mouse Hashtag 3	BioLegend	155835
TotalSeq^™^-B0304 anti-mouse Hashtag 4	BioLegend	155837
TotalSeq^™^-B0305 anti-mouse Hashtag 5	BioLegend	155839
TotalSeq^™^-B0306 anti-mouse Hashtag 6	BioLegend	155841
TotalSeq^™^-B0307 anti-mouse Hashtag 7	BioLegend	155843
TotalSeq^™^-B0308 anti-mouse Hashtag 8	BioLegend	155845
TotalSeq^™^-B0309 anti-mouse Hashtag 9	BioLegend	155847

## Highlights

- Vivo-seq captures integrated transcriptional and intracellular signaling information
- DES fixation preserves morphology, PTMs, and enables long-term intracellular analysis
- Simultaneous ERK1/2 and c-FOS phosphorylation synergizes to induce IL-2 in Th17 cells
- Early IL-2 production in Th17 cells enhances stability or plasticity upon re-stimulation
