# Supplementary material for: Integration of phospho-signaling and transcriptomics in single cells reveals distinct Th17 cell fates
Source: Cell Rep. Author manuscript; Available in PMC 2025 Oct 31. (PMC12577753; doi:10.1016/j.celrep.2025.116006)
Supplement: 1 [file NIHMS2107240-supplement-1.pdf]

Cell Reports, Volume *44*

Supplemental information

Integration of phospho-signaling and transcriptomics in single cells reveals distinct Th17 cell fates

Seth D. Fortmann, Awalpreet S. Chadha, Blake F. Frey, Asif Elahi, Vidya Sagar Hanumanthu, Shanrun Liu, Andrew Goldsborough, P. Brent Ferrell Jr., Maria B. Grant, Casey T. Weaver, and Robert S. Welner

SUPPLEMENTARY MATERIALS

Integration of Phospho-Signaling and Transcriptomics in Single Cells Reveals Distinct Th17 Cell Fates

SUPPLEMENTAL FIGURES

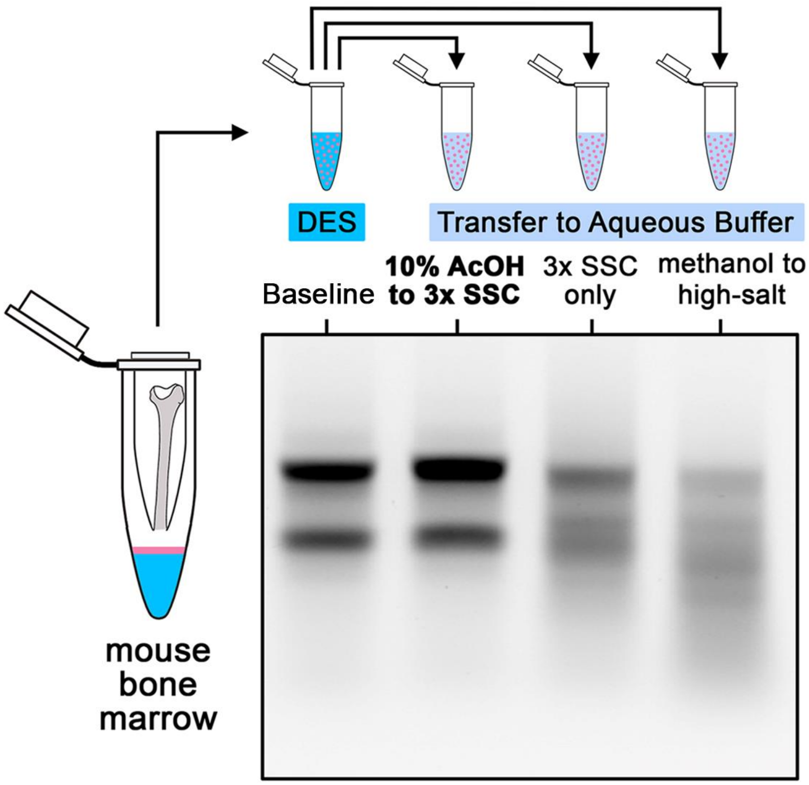

Supplemental Figure 1. RNA quality after transfer from DES to aqueous buffer. Gel electrophoresis for RNA integrity from mouse bone marrow fixed with DES before and after transfer to aqueous buffer using three different methods: (1) 10% AcOH in DES for 5 minutes at RT followed by excess 3x SSC, (2) excess 3x SSC alone, or (3) methanol followed by high salt buffer [4M ammonium sulfate]. The same bone marrow sample was used for each step.

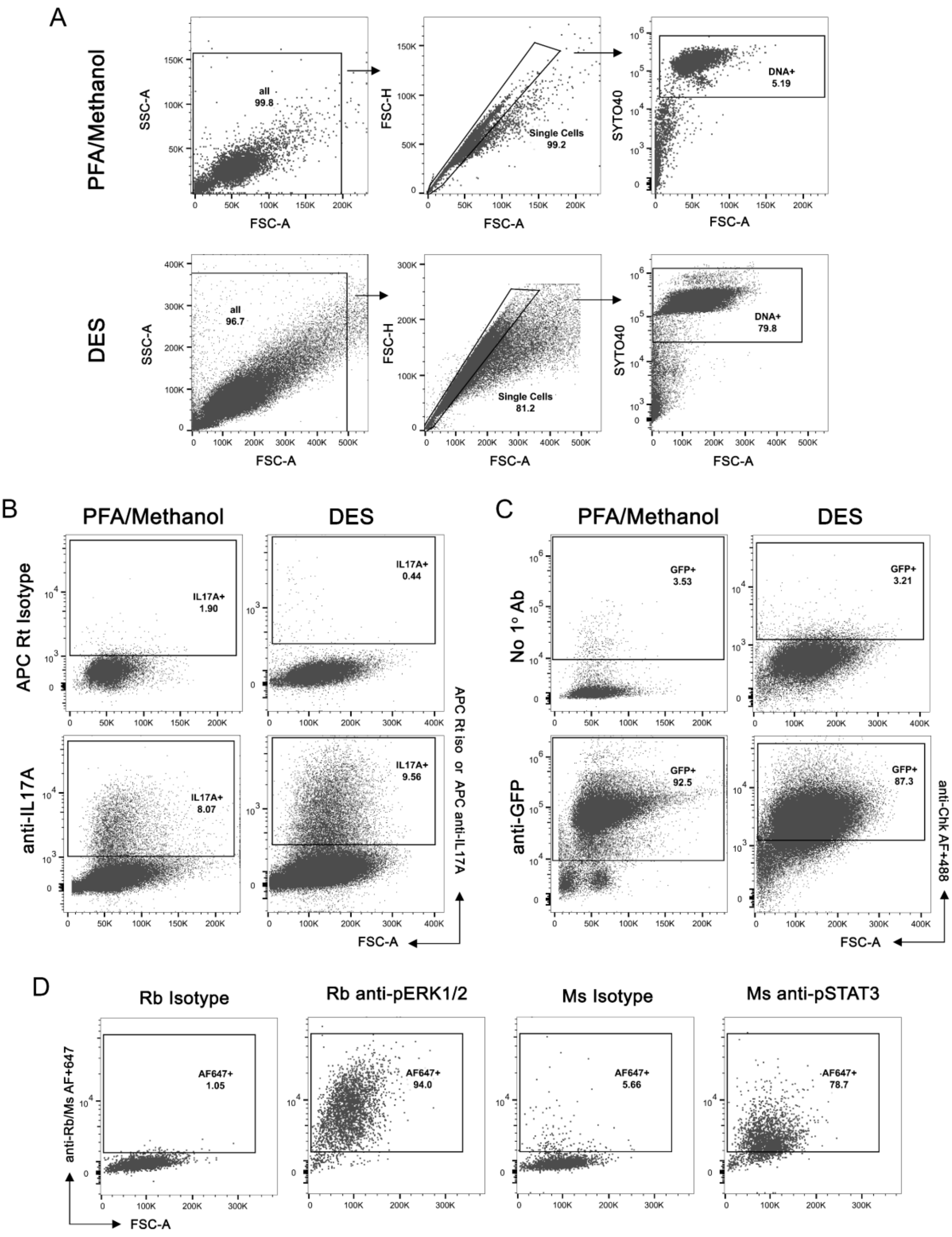

Supplemental Figure 2. Flow cytometry gating scheme and isotype controls for PFA/methanol and DES-fixed Th17 T cells. (**A**) Gating scheme for Th17 T cells fixed with either PFA/methanol (top) or DES (bottom). (**B**) IL-17A isotype controls for PFA/methanol and DES fixed cells. (**C**) anti-GFP negative controls for PFA/methanol and DES fixed cells. (**D**) Rabbit and mouse isotype controls for phospho-flow cytometry in DES fixed cells.

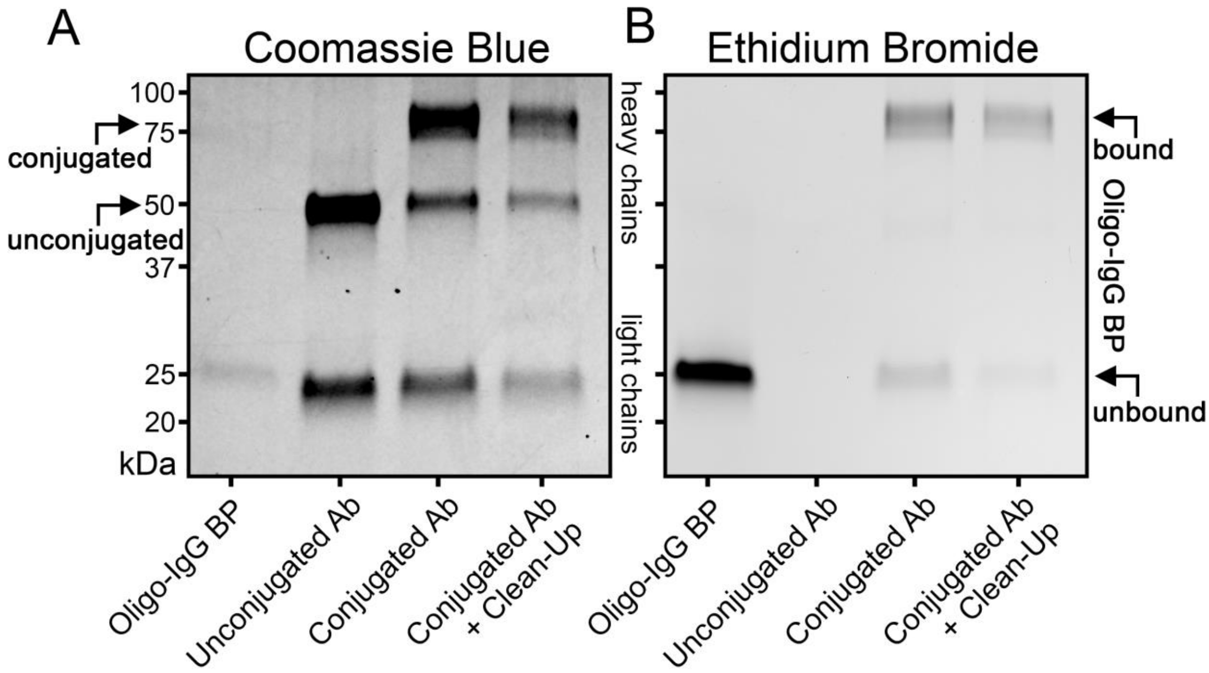

Supplemental Figure 3. Antibody-oligonucleotide conjugation for intracellular CITE-seq. (**A**) Polyacrylamide gel electrophoresis under denaturing conditions stained with Coomassie blue showing antibody conjugation. Each lane = 1μg of antibody. (**B**) The same gel from (A) stained with ethidium bromide showing bound and unbound oligonucleotide before and after clean-up with human IgG Fc magnetic beads.

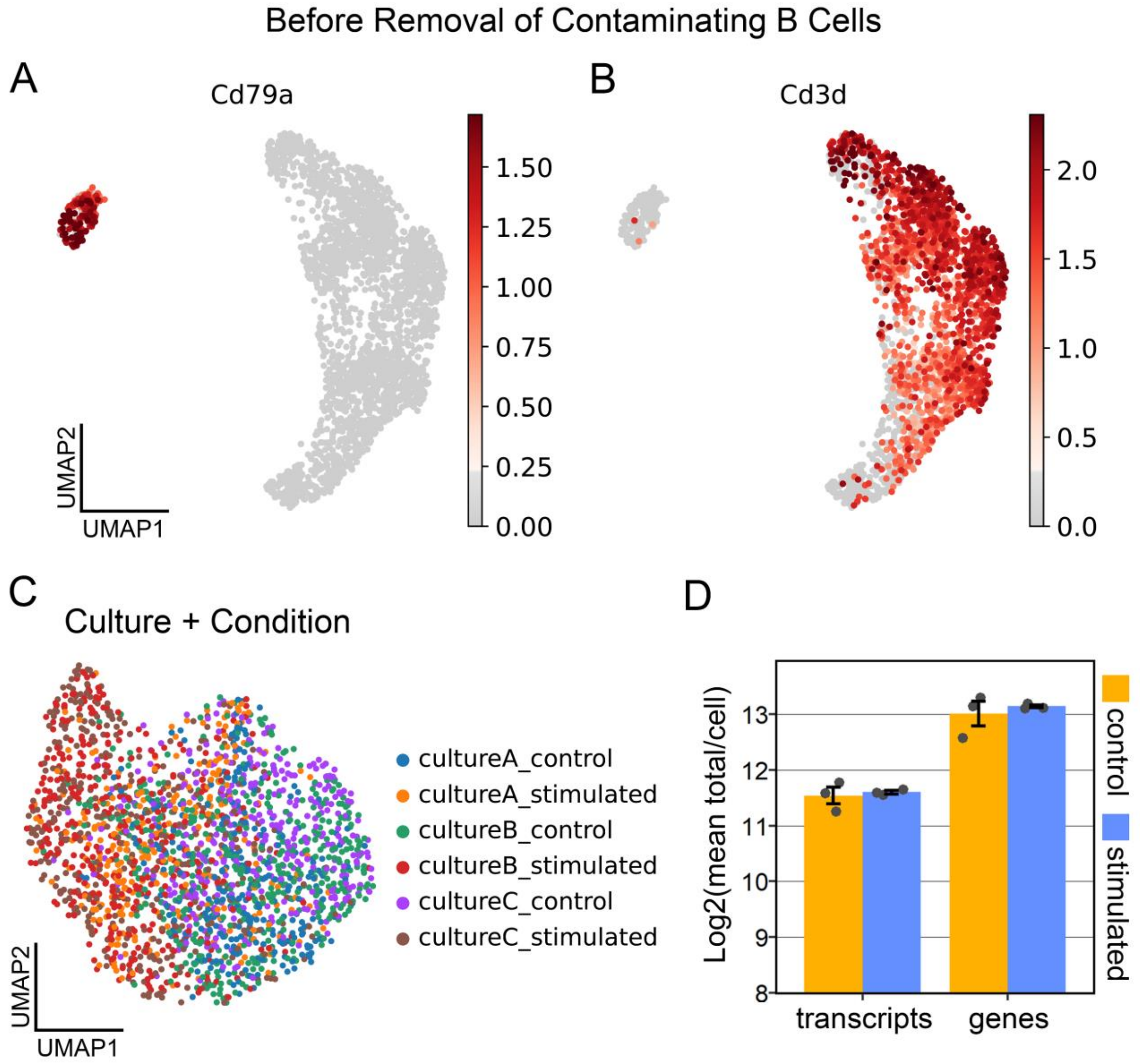

Supplemental Figure 4. Th17 T cell single-cell RNA-sequencing. Normalized expression of (**A**) Cd79a and (**B**) Cd3d in UMAP space showing contaminating B cells. (**C**) UMAP showing integration of cultures and conditions. (**D**) Mean total genes and total transcripts for each culture/condition (n=6).

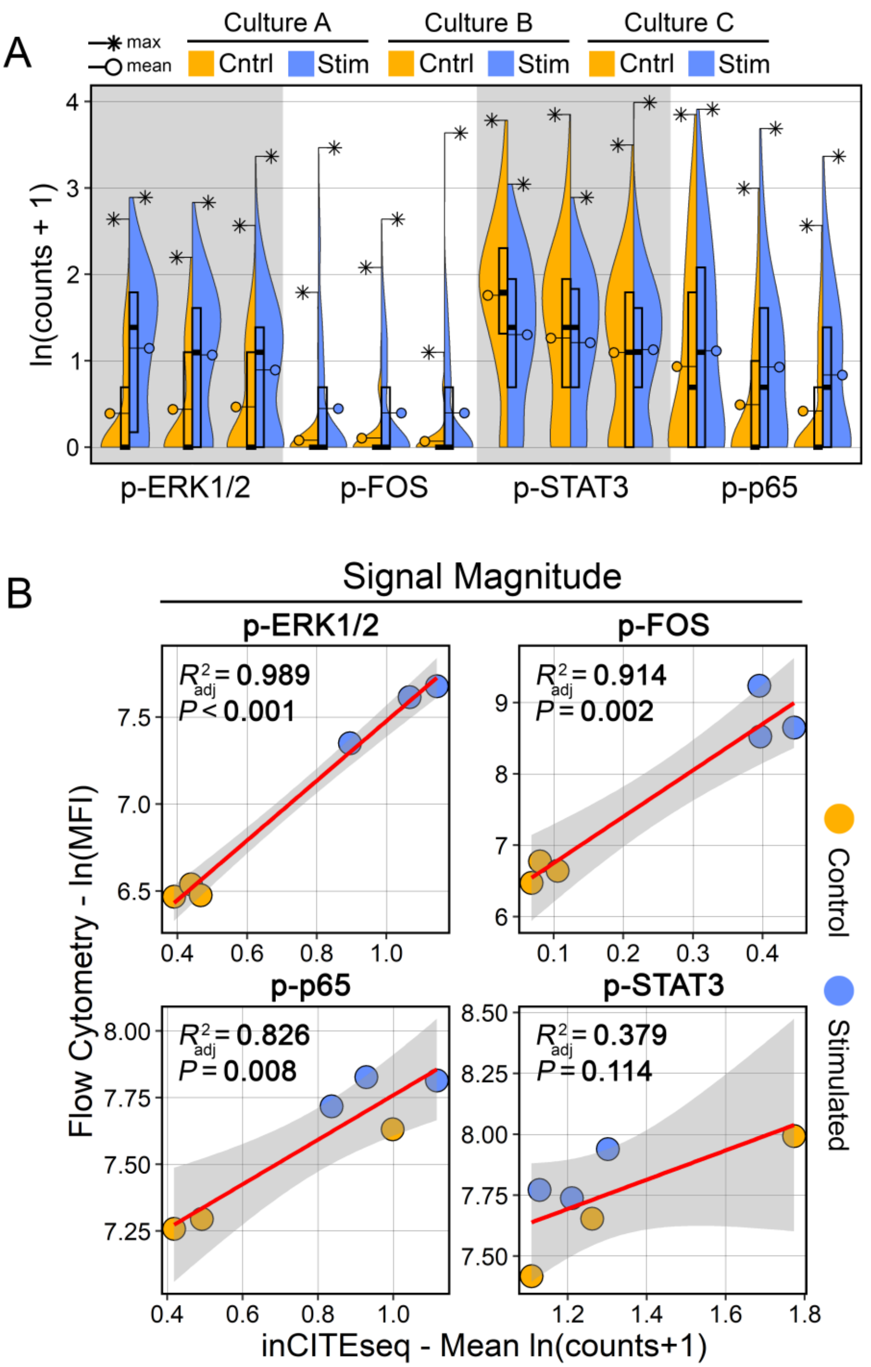

Supplemental Figure 5. Intracellular CITE-seq signal magnitude compared to paired flow cytometry for four phospho-targets during Th17 stimulation. (**A**) Comparisons of log normalized counts showing stimulated versus control for each of the 4 phospho-targets. Split violins represent paired cultures. (**B**) Linear regressions for the 4 phospho-targets comparing signaling magnitude from inCITE-seq (ln(counts-isotype +1)) versus flow cytometry (mean fluorescent intensity). The dynamic ranges of inCITE-seq compared to flow cytometry measurements for p-ERK1/2, p-FOS, p-STAT3, and p-p65 under stimulated and control conditions are as follows: p-ERK1/2 control (flow cytometry, min:0, max:12552.3, median:602,

mean:721.9; inCITE-seq, min:0, max:13, median:0, mean:1), p-ERK1/2 stimulated (flow cytometry, min:0, max:53503, median:1703.6, mean:2495.4; inCITE-seq, min:0, max:28, median:2, mean:2.7), p-FOS control (flow cytometry, min:0, max:41437.6, median:746.1, mean:897.9; inCITE-seq, min:0, max:7, median:0, mean:0.2), p-FOS stimulated (flow cytometry, min:0, max:350271, median:6516.4, mean:10124.7; inCITE-seq, min:0, max:37, median:0, mean:1.1), p-STAT3 control (flow cytometry, min:0, max:54930, median:2562.4, mean:3183.2; inCITE-seq, min:0, max:56, median:3, mean:5), p-STAT3 stimulated (flow cytometry, min:0, max:27118.1, median:2908, mean:3179.5; inCITE-seq, min:0, max:53, median:3, mean:3.5), p-p65 control (flow cytometry, min:0, max:40100.7, median:1663.1, mean:2505.6; inCITE-seq, min:0, max:204, median:0, mean:2.5), and p-p65 stimulated (flow cytometry, min:0, max:39454.4, median:2180.5, mean:3531.9; inCITE-seq, min:0, max:49, median:1, mean:3.3). MFI, mean fluorescent intensity.

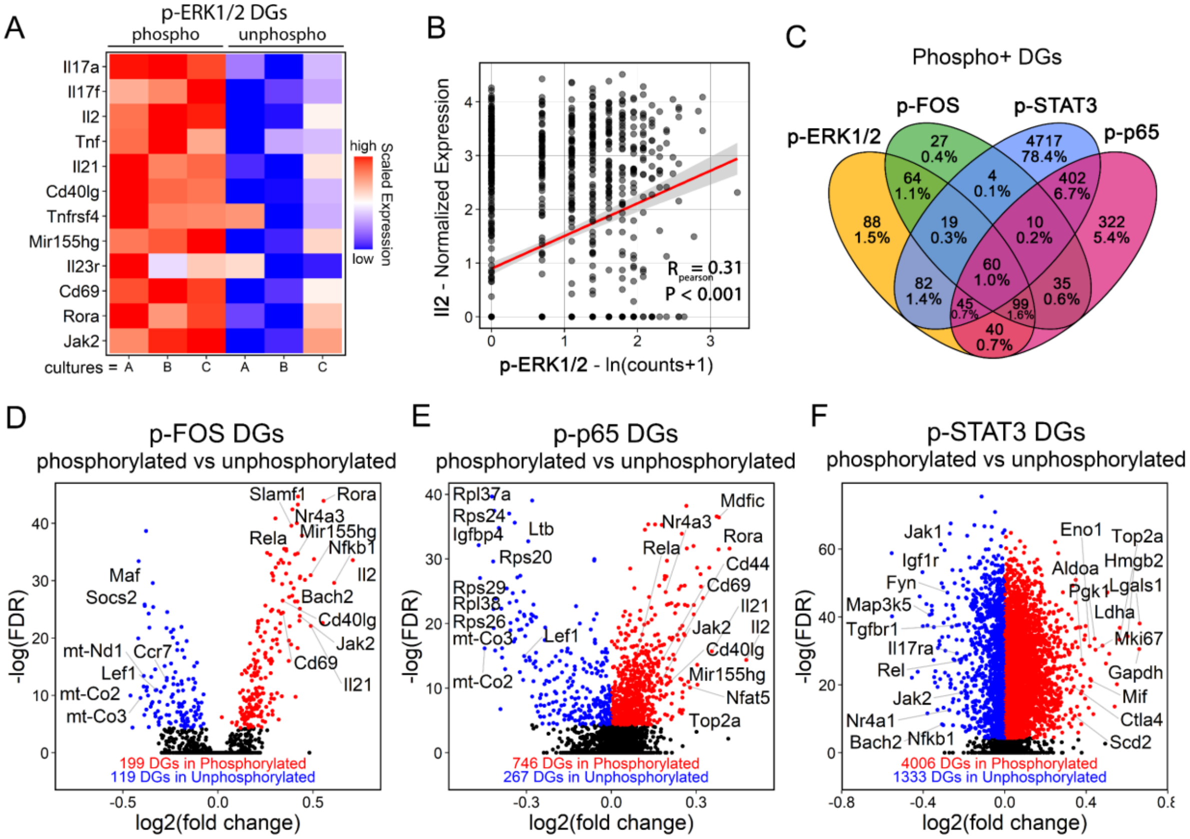

Supplemental Figure 6. Extended data from Th17 intracellular CITE-seq. (**A**) Heatmap showing scaled expression of selected p-ERK1/2 differentially expressed genes (DGs) by culture/condition. (**B**) Scatter plot showing Pearson correlation between IL2 expression and p-ERK1/2 magnitude. (**C**) 4-way Venn diagram showing overlap in DGs for p-ERK1/2, p-FOS, p-STAT3, and p-p65. (**D**,**F**) Volcano plots showing selected DGs for phosphorylated versus unphosphorylated (**D**) p-FOS, (**E**) p-p65, and (**F**) p-STAT3.

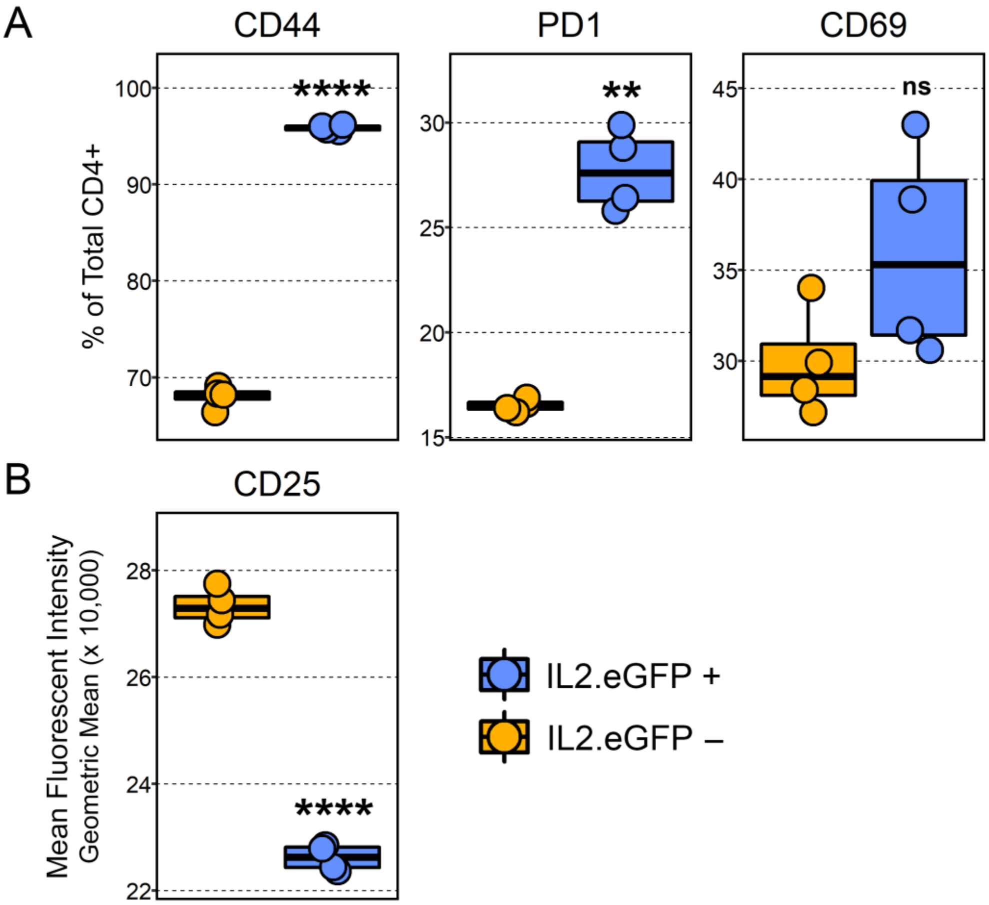

Supplemental Figure 7. IL2 production correlates with increased T-cell activation. (A-B) Bar plots (n=4 independent cultures) showing percentage positivity **(A)** of CD44, PD-1, and CD69, and MFI **(B)** of CD25 in IL.2eGFP- (yellow) versus IL2.eGFP+ (blue) cells sorted at 40 hours following Th17 polarization. Student's t test: ns, * p ≤ 0.05; ** p ≤ 0.01; *** p ≤ 0.001; **** p ≤ 0.0001. Data are represented as mean +/- SEM.

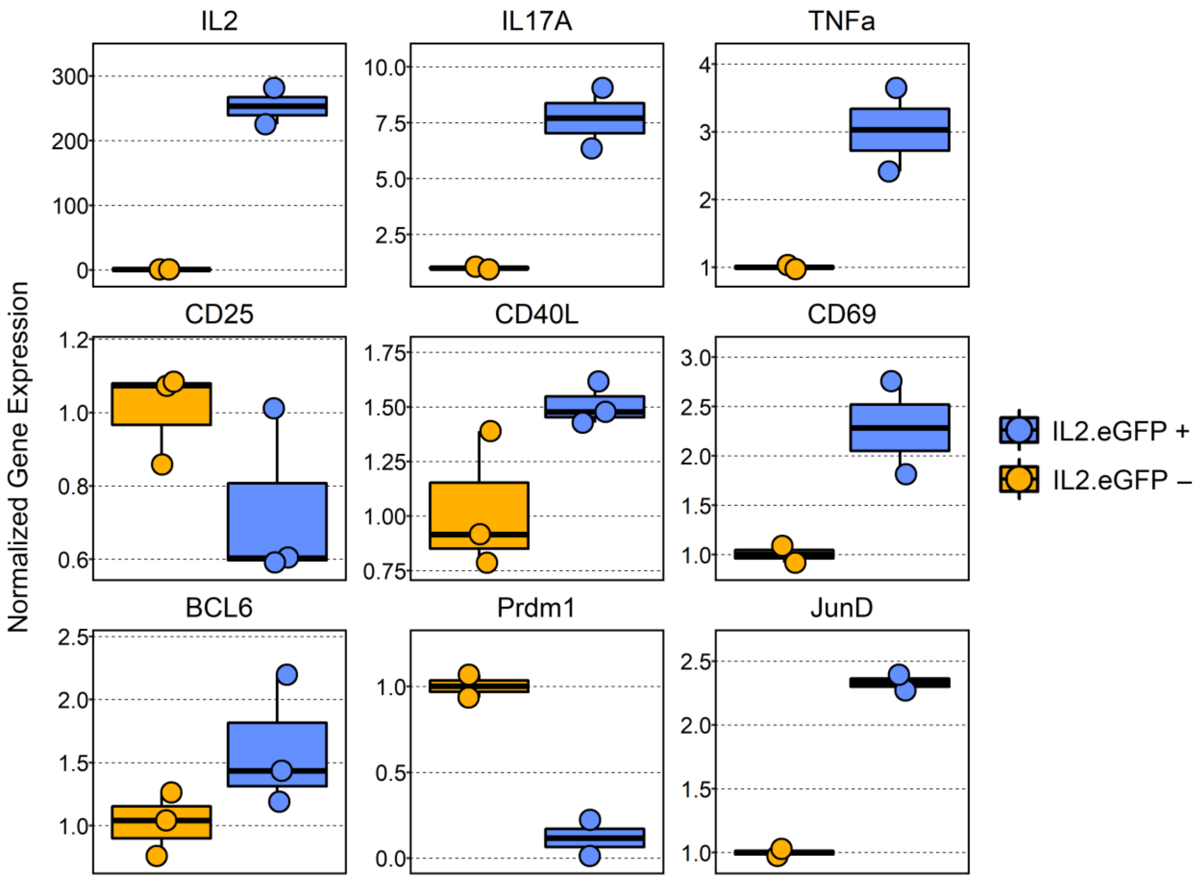

Supplemental Figure 8. Validation of selected activation associated genes in early IL-2-producing Th17 Cells. RNA was isolated from IL.2eGFP- (yellow) versus IL2.eGFP+ (blue) cells sorted at 40 hours following Th17 polarization (n=3 independent experiments) and PCR was performed for selected genes.

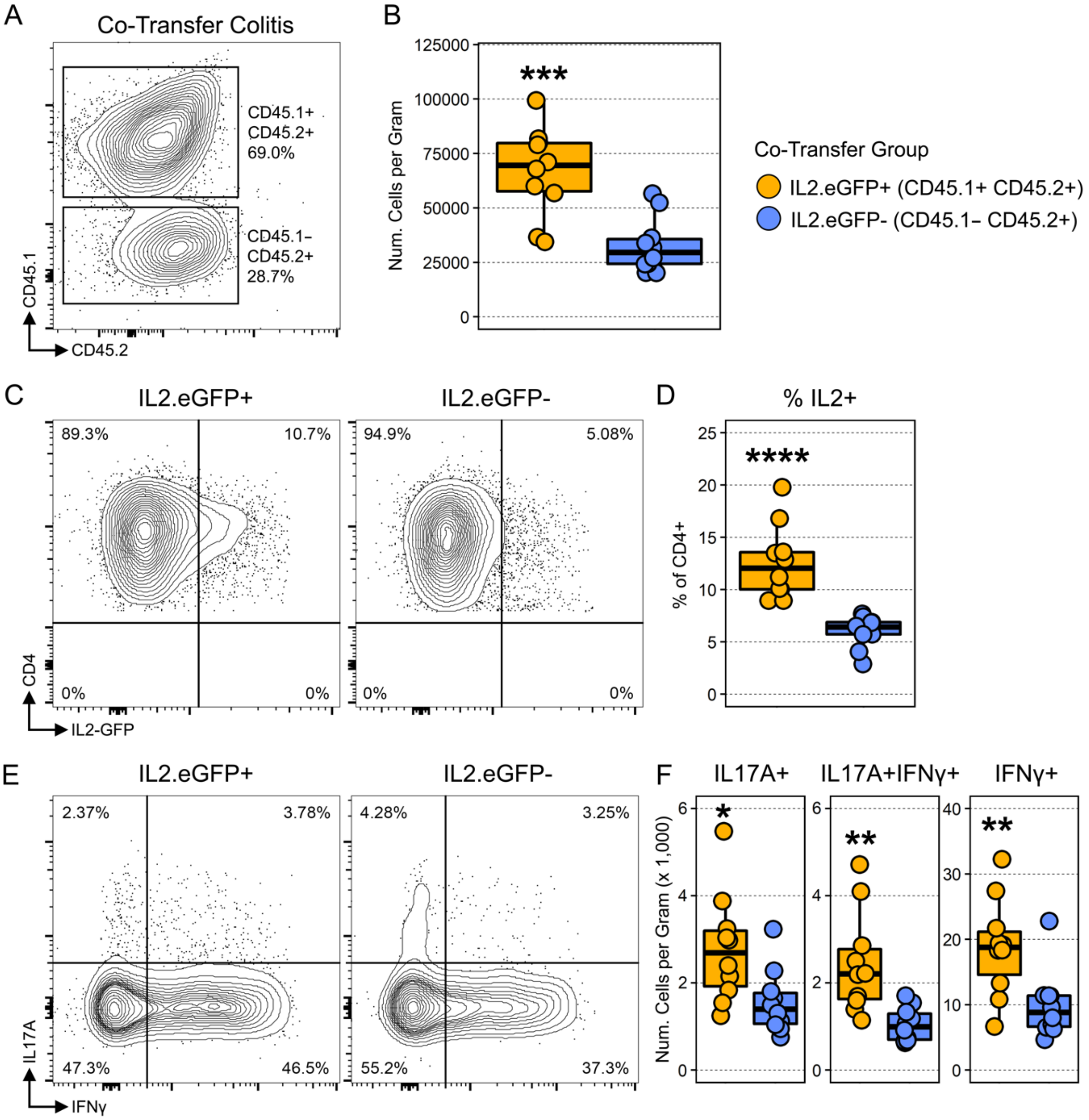

Supplemental Figure 9. Ex vivo validation linking early T-cell activation with subsequent cytokine production and increased Th17 transdifferentiation. (A) Flow cytometry plot showing percentage of IL-2+ CD45.1/45.2+ and IL-2- CD45.2+ cells retrieved from the colonic lamina propria on day 14 following co-transfer (n=10 animals). **(B)** Box plots showing the number of cells per gram of colon. **(C-D)** Flow cytometry plots and box plot showing percentage of IL-2 eGFP+ CD4+ T cells within the recovered IL-2+ CD45.1/45.2+ (left/yellow) and IL-2- CD45.2+ (right/blue) T cell populations. (E) Flow cytometry plot showing expression of IL-17A and IFNγ in the transferred IL-2+ CD45.1/45.2+ (left) and IL-2- CD45.2+ (right) cells. (F) Box plots showing the number of IL17A+ , IL17A+ IFNγ+ and IFNγ+ cells per gram of colon. One-way ANOVA with Holm-Sidak post-hoc test. * $p \leq 0.05$; ** $p \leq 0.01$; *** $p \leq 0.001$; **** $p \leq 0.0001$. Data are represented as mean +/- SEM.
